# Maternal serum vitamin D levels and preeclampsia among Kenyan pregnant women: A matched case-control study

**DOI:** 10.1371/journal.pone.0358781

**Published:** 2026-09-22

**Authors:** Kimbley Asaso Omwodo, Joy Njoki Kimani, Kuboi Nicodemus, David Mbugua Kioko, Antoniya Georgieva

**Affiliations:** 1 Department of Obstetrics and Gynaecology, The Nairobi Hospital, Nairobi, Kenya; 2 Department of Reproductive Health, Moi University, College of Health Sciences (MUCHS), Eldoret, Kenya; 3 Department of Psychology, Eldoret Oncology Associates, Eldoret, Kenya; 4 Department of Medical Oncology, Eldoret Oncology Associates, Eldoret, Kenya; 5 Nuffield Department of Women's & Reproductive Health, University of Oxford, Level 3 Women's Centre, John Radcliffe Hospital, Oxford, United Kingdom; Norwegian Institute of Public Health: Folkehelseinstituttet, NORWAY

## Abstract

**Background:**

Preeclampsia accounts for about one in five direct maternal deaths in Kenya. Low vitamin D levels have been linked to its pathophysiology. Despite this, context‑specific data from African populations remain limited. The study aimed to determine the association between maternal serum 25-hydroxyvitamin D [25(OH)D] concentrations (nmol/L) in pregnancy and the risk of preeclampsia in a Kenyan population.

**Methods:**

A matched case-control study was conducted at Moi Teaching and Referral Hospital, Kenya. A total of 118 women with confirmed viable intrauterine pregnancies at ≥28 weeks’ gestation were analysed. Fifty-nine women with preeclampsia (cases) and 59 controls were matched by parity, gestation, and age. Serum 25(OH)D was quantified. Vitamin D status was categorised as suboptimal (<50 nmol/L) or sufficient (≥50 nmol/L) status. The association between vitamin D status and preeclampsia was assessed using conditional logistic regression. Odds ratios (ORs) with 95% confidence intervals (CIs) were presented.

**Results:**

Median serum 25(OH)D was significantly lower in preeclampsia cases compared with controls [67.1 nmol/L (interquartile range 47.8–84.0) vs. 77.6 nmol/L (62.4–99.7); *P* = 0.001]. Suboptimal vitamin D levels (<50 nmol/L) were observed in 32.2% of preeclampsia cases versus 6.8% of controls (*P* = 0.002). In conditional logistic regression accounting for matching, women with suboptimal vitamin D had higher odds of preeclampsia (OR 8.0; 95% CI: 1.84–34.78; *P* = 0.006). Each 10 nmol/L decrease in serum 25(OH)D was associated with a 29% increase in preeclampsia odds (OR 1.29; 95% CI: 1.09–1.54; *P* = 0.004).

**Conclusions:**

In this study, suboptimal maternal serum vitamin D status demonstrated a significant association with preeclampsia. There is potential value in antenatal screening for vitamin D status. Findings support further research into vitamin D supplementation as a preventive intervention for preeclampsia.

**Trial registration:**

Pan African Clinical Trial Registry (PACTR202505516303679).

## Introduction

Hypertensive disorders of pregnancy (HDP) refer to a group of medical disorders characterised by high blood pressure occurring during pregnancy. HDP affects up to 10% of pregnancies worldwide [1]. According to the National High Blood Pressure Education Program Working Group on High Blood Pressure in Pregnancy, HDP is classified into four categories: chronic hypertension, preeclampsia (and related disorders: eclampsia and haemolysis, elevated liver enzymes, low platelets [HELLP] syndrome), preeclampsia superimposed on chronic hypertension and gestational hypertension [2].

In Kenya, approximately 1 in 5 direct maternal deaths result from hypertensive disorders, including preeclampsia [3].

Preeclampsia (PE) aetiology can be divided into two distinct phases. The first phase is characterised by defective placentation. The progression into the second phase is marked by decreased vascularisation occurring at the placental site, which initiates a maternal inflammatory response. PE modifies angiogenic as well as antiangiogenic factors, e.g., serum placental growth factor (PIGF), soluble fms-like tyrosine kinase 1 (sFlt-1), and soluble endoglin (sENG) [1].

Vitamin D is a family of chemically related secosteroid hormones. It is synthesised primarily in the skin following exposure to UVB radiation from sunlight (wavelength, 290–315 nm). Solar ultraviolet B radiation infiltrates the skin and converts 7-dehydrocholesterol into provitamin D3, which is quickly transformed into vitamin D3. Vitamin D from both cutaneous synthesis and dietary intake is metabolised in the liver into 25-hydroxyvitamin D (25(OH)D), a vitamin D status biomarker, and into 1,25-dihydroxyvitamin D (1,25(OH)_2_D). Although no consensus has been reached on vitamin D status definitions, the Endocrine Society mentions serum 25(OH)D < 20 ng/mL (50 nmol/L) as a common threshold in studies [4]. In contrast, the Institute of Medicine (IOM) defines deficiency as concentrations below 12 ng/mL (30 nmol/L) and insufficiency as <20 ng/mL (<50 nmol/L) [5]. Pregnant women are a population at increased risk of vitamin D deficiency. Factors such as inadequate intake of vitamin D, high Body Mass Index (BMI), and a poor-quality diet further heighten this risk.

Several mechanisms can elucidate the protective effects of vitamin D in PE. Vitamin D modulates pro-inflammatory responses and also contributes to blood pressure reduction through the renin-angiotensin system (RAAS) [6,7].

A systematic review in 2020 that included pooled data from twenty-seven randomised controlled trials (RCTs) involving 4,777 participants reported that vitamin D supplementation resulted in a 63% risk reduction in the occurrence of PE [8]. Even so, the studies included in the review did not strictly select participants on the basis of vitamin D status, nor did they evaluate the achievement of optimum vitamin D levels post-supplementation. The authors also noted that the results could not be generalised to an African population, given the absence of studies that included this cohort. A more recent meta-analysis of 33 RCTs (n = 10,613) similarly reported a 45% reduction in preeclampsia risk with supplementation [9]. Notably, the analysis included only one study from Africa (Congo).

Genetic and phenotypic diversity in vitamin D metabolism has been observed among different races, with individuals of European ancestry exhibiting higher vitamin D concentrations [10]. Skin pigmentation negatively influences vitamin D synthesis, as melanin acts as an absorbent filter (scattering UVR), reducing the efficiency of vitamin D production by UV induction and subsequent circulating levels of 25(OH)D [11,12]. Mogire et al. observed that many African countries lacked studies measuring vitamin D levels [13].

This study aimed to ascertain the proportion of vitamin D deficiency among parturients with viable pregnancies and to compare the vitamin D levels between women with and without pre-eclampsia. By generating context-specific evidence, the study results would provide insight into whether antenatal vitamin D screening warrants further investigation (as a potential component of PE risk stratification) in Kenya.

**Objectives:**

1. To describe the proportion of vitamin D deficiency among the study sample of women with viable pregnancy at the Moi Teaching and Referral Hospital (MTRH).
2. To compare vitamin D status among pregnant women with and without PE.

## Methods

### Study design and setting

We conducted a single-centre matched case-control study at MTRH in Eldoret, Kenya (elevation 2,100 metres above sea level). MTRH is a public multi-speciality tertiary healthcare facility with about 12,000 deliveries annually. The referral population includes the western Kenya region, parts of eastern Uganda, southern Sudan, northern Tanzania, and the Democratic Republic of Congo (DRC). Participant recruitment spanned from 1 August 2025 to 31 January 2026. Both dry (August to October) and wet (November to January) seasons were covered to mitigate seasonal variation in vitamin D status.

Eligible participants were screened consecutively and enrolled upon admission to the MTRH antenatal ward (ANW) or labour ward (LW) unit. Maternal serum 25(OH)D was measured at enrolment. Data collection occurred concurrently with recruitment. Research was conducted in accordance with the protocol previously published [14]. The Strengthening the Reporting of Observational Studies in Epidemiology (STROBE) guidelines were used in manuscript preparation. The completed STROBE checklist is provided as **S1 File**.

### Study size

The study was conceptualised as a pilot investigation to generate preliminary context-specific evidence on vitamin D status and PE in a Kenyan population. Based on prior African studies [13]; fifty-nine case-control pairs would provide 80% power to detect a 25-percentage-point difference in deficiency prevalence between groups, using a two-proportion comparison at α = 0.05. The target sample size was inflated to 120 participants to account for potential attrition.

### Participants

Cases were pregnant women with viable pregnancies (≥28 weeks gestation) diagnosed with PE. Controls comprised normotensive pregnant women (blood pressure <140/90 mmHg without proteinuria). They were concurrently recruited from the same hospital wards and matched to cases by maternal age (±3 years), gestational age (±1 week), and parity (0, 1–3, ≥ 4). The selected matching factors were established risk factors for PE and potential confounders of the association between vitamin D status and PE [15,16].

Ascertainment of cases and controls was performed by trained research assistants who conducted daily reviews of ward admission registers. This was done to ensure standardised diagnostic verification using the facility protocols. Hospital-based controls supported comparable healthcare access.

### Eligibility criteria

Inclusion criteria were (a) maternal age of ≥ 18 years at the time of consent, (b) confirmed singleton pregnancy of 28 weeks gestation or greater (confirmed by ultrasound), (c) delivery at MTRH, and (d) ability to provide written informed consent.

Exclusion criteria included (a) pre-existing chronic kidney disease, (b) pre-existing parathyroid conditions, (c) those on cardiac medication, (d) pre-existing thrombophilia, (e) participating in a similar interventional study.

### Definitions

PE was defined prospectively according to the guidelines of the International Society for the Study of Hypertension in Pregnancy [17]; i.e., new-onset hypertension, defined as systolic blood pressure of 140 mmHg or greater and/or diastolic blood pressure of 90 mmHg or greater (based on an average of two measurements) in a patient who was previously normotensive, accompanied by at least one other symptom and sign related to PE. These symptoms and signs include:

- Proteinuria (≥300 mg/24 hours or more than 0.3 g/day),
- Acute kidney injury (creatinine levels at or greater than 90 μmol/L),
- Liver involvement (elevated transaminases, for example, ALT or AST exceeding 40 IU/L), Neurological symptoms (such as altered mental status, blindness, stroke, severe headaches, persistent visual scotomata),
- Haematological abnormalities (including thrombocytopenia, i.e., platelet count below 150,000/μL, disseminated intravascular coagulation, and haemolysis),
- Cardiorespiratory complications (pulmonary oedema, myocardial ischaemia or infarction, oxygen saturation below 90%, use of 50% or more inspired oxygen for over an hour, intubation not related to caesarean delivery), or
- Uteroplacental dysfunction (foetal growth restriction, angiogenic imbalance, placental abruption) occurring after 20 weeks gestation

Vitamin D status was categorised based on Institute of Medicine (IOM) guidelines [5]. Categories were deficient (<30 nmol/L), insufficient (30–49 nmol/L), and sufficient (≥50 nmol/L). The Endocrine Society notes that vitamin D status of < 50 nmol/L is associated with increased risk of hypertensive disorders of pregnancy [4]. Similar to Gidlof et al., categorisation for analysis was also done according to suboptimal (<50 nmol/L) versus sufficient (≥50 nmol/L) status [18].

**Covariates:** Demographic factors, including maternal age, parity, gestational age, and pre-pregnancy BMI, were collected. Gestational age was calculated from the first day of the last menstrual period. Parity was defined as the number of times the participant has given birth to a foetus of ≥ 24 weeks gestational age, regardless of pregnancy outcome. BMI was calculated as weight in kilograms divided by height in metres squared (kg/m^2^). Participant and medical files reporting relevant medical diagnoses (chronic hypertension, diabetes) were also collected. Data were collected using structured interviewer-administered questionnaires.

**Biological sampling and assessment of serum vitamin D:** Approximately 5 mL of maternal blood was obtained via venipuncture into serum separator tubes (Becton Dickinson, Franklin Lakes, NJ, USA). Laboratory technicians processing the samples were blinded to case/control status. Samples were allowed to clot at room temperature for 30 minutes, then placed in an insulated cool box for transportation to the Cerba Lancet laboratory (ISO15189 accredited) for processing. The transportation temperature of the cool box was maintained at 2–8°C. The transportation took place within 2 hours. Serum 25(OH)D levels (combined D2 and D3) were quantified using High-Performance Liquid Chromatography (HPLC). A photodiode array detector (ClinRep HPLC Complete Kit, Recipe, München, Germany) was used. The Cerba Lancet laboratory participates in the Vitamin D External Quality Assessment Scheme (DEQAS) [19].

### Bias

Patients with pre-existing conditions affecting vitamin D metabolism were excluded to reduce selection bias (i.e., chronic kidney disease, parathyroid conditions and thrombophilia).

Measurement bias was reduced by laboratory technicians processing vitamin D assays being blinded to case/control status.

Matching study design and conditional logistic regression (at analysis) addressed confounding.

### Statistical analysis

Data analysis was done using Stata software version 19.0 (StataCorp LLC, College Station, TX, USA). Normality of continuous variables was assessed using the Kolmogorov-Smirnov test. Baseline characteristics were summarised as means with standard deviations (SD) or medians with interquartile ranges (IQR) for continuous variables as appropriate. Categorical variables were presented as frequencies with percentages. Baseline characteristics were summarised and compared by PE status. Differences in continuous variables were compared using paired t-tests for normally distributed continuous variables, Wilcoxon signed-rank tests for skewed continuous variables, and McNemar's test or Fisher's exact test for categorical variables. Matched study design was accounted for.

Vitamin D status was categorised a priori [4,5]. Conditional logistic regression (to account for the matching factors) was used to assess the association between vitamin D status and PE. Dose–response analysis was performed treating serum 25(OH)D as a continuous variable. Missing data were handled by complete case analysis; matched pairs with missing covariate data in either member were excluded from adjusted models. Significance was set at p-value < 0.05 (two-sided).

### Ethical considerations

The study protocol has been previously published [14] and prospectively registered as an observational study with the Pan African Clinical Trial Registry (PACTR). Registration number PACTR202505516303679. Review and ethical approval were obtained from the Institutional Review and Ethics Committee of Moi University/MTRH (IREC approval number 0005019). Written authorisation was obtained from the management of MTRH to conduct the study (reference ELD/MTRH/R&P/10/2/V.2/2010). A research licence was also granted from the National Commission for Science, Technology, and Innovation (NACOSTI) Kenya before commencement of the study (Licence number NACOSTI/P/25/417821). Written informed consent was taken from each participant before enrolment into the study. Autonomy was upheld by providing participants all the necessary information and freedom to withdraw from the study at any point without need for justification.

## Results

### Participant characteristics and vitamin D status

Between August 2025 and January 2026, 135 women with pregnancies ≥28 weeks were screened for eligibility. Five did not meet inclusion (3 had parathyroid conditions, 2 had chronic kidney disease) and 6 declined participation. Reasons for declining included anxiety about needles and excessive blood loss (3) and perceived lack of direct benefit (2). One woman in labour declined due to competing clinical priorities.

All 124 women meeting eligibility were enrolled into the study. Six enrolled participants (3 cases, 3 controls) were excluded post-hoc because no eligible 1:1 match could be identified. Excluded participants did not differ systematically from included participants by demographic characteristics or vitamin D status (S2 File).

**Fig 1:**
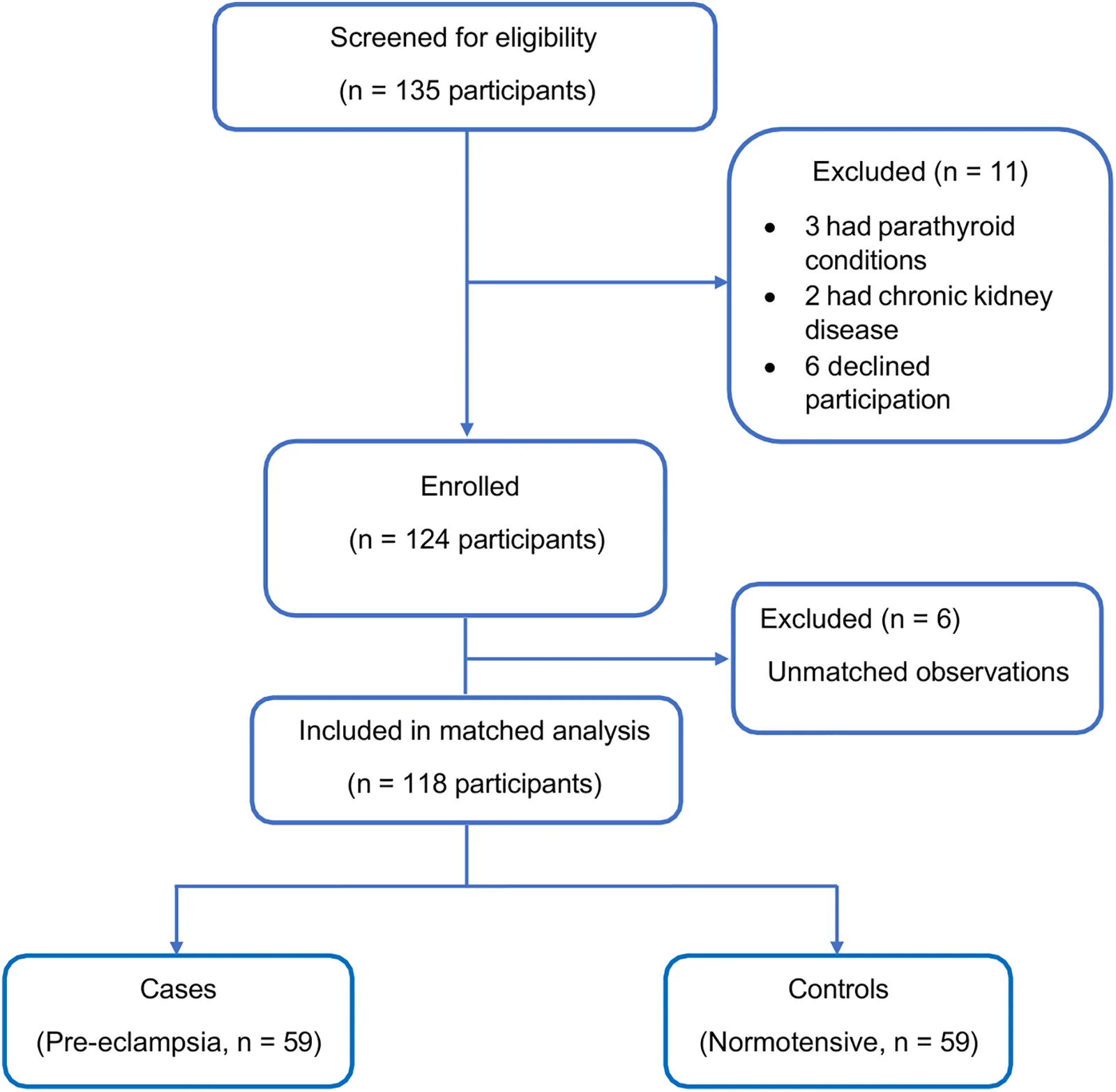
Study flow diagram showing participant selection. Matching was performed on maternal age (±3 years), parity (0, 1–3, ≥ 4), and gestational age at delivery (±1 week).

The final analytic sample consisted of 118 participants (59 matched case-control pairs) (Fig 1).

Matching achieved comparability for mean maternal age, gestational age and parity. Sociodemographic factors were also similarly distributed between groups. Women with PE were more likely to be referred from peripheral facilities compared to controls (78.0% vs. 39.0%; *P* < 0.001). This reflects the referral nature of the cases in this study group. Mean maternal serum 25(OH)D concentrations were lower among women with PE compared to controls (64.3 ± 24.5 nmol/L vs. 82.5 ± 28.8 nmol/L). Baseline characteristics by PE status are presented in Table 1.

**Table 1:** Baseline maternal characteristics by preeclampsia status.

Characteristic	Total (n = 118)	Controls (n = 59)	Preeclampsia Cases (n = 59)	*P*-value
**Maternal age (years), mean (SD)**	28.9 (6.2)	28.6 (5.8)	29.3 (6.7)	0.067‡
**Gestational age at delivery (weeks), mean (SD)**	35.4 (3.6)	35.6 (3.4)	35.1 (3.8)	0.433‡
**Level of education, n (%)**				0.612*
No formal schooling	3 (2.5)	1 (1.7)	2 (3.4)	
Primary completed	22 (18.6)	10 (16.9)	12 (20.3)	
Secondary completed	42 (35.6)	24 (40.7)	18 (30.5)	
College/University completed	51 (43.2)	24 (40.7)	27 (45.8)	
**Work status, n (%)**				0.991*
Homemaker	9 (7.6)	4 (6.8)	5 (8.5)	
Non-government employee	12 (10.2)	7 (11.9)	5 (8.5)	
Self-employed	20 (16.9)	9 (15.3)	11 (18.6)	
Student	7 (5.9)	3 (5.1)	4 (6.8)	
Unemployed (able to work)	63 (53.4)	29 (49.2)	34 (57.6)	
Unemployed (unable to work)	7 (5.9)	7 (11.9)	0 (0.0)	
**Marital status, n (%)**				0.853*
Cohabitating	2 (1.7)	1 (1.7)	1 (1.7)	
Currently married	97 (82.2)	48 (81.4)	49 (83.1)	
Divorced	2 (1.7)	0 (0.0)	2 (3.4)	
Never married	17 (14.4)	10 (16.9)	7 (11.9)	
**Parity category, n (%)**				
0	25 (21.2)	12 (20.3)	13 (22.0)	
1–3	79 (66.9)	40 (67.9)	39 (66.1)	
≥4	14 (11.9)	7 (11.9)	7 (11.9)	
**Antenatal care visits, n (%)**				0.987*
0–3 visits	47 (39.8)	23 (39.0)	24 (40.7)	
4–7 visits	59 (50.0)	28 (47.5)	31 (52.5)	
≥8 visits	12 (10.2)	8 (13.6)	4 (6.8)	
**Referred from peripheral facility, n (%)**	69 (58.5)	23 (39.0)	46 (78.0)	**<0.001**¶
**BMI in early pregnancy (kg/m²), mean (SD)**				0.319‡
*n*	*114*	*57*	*57*	
	26.4 (5.4)	25.9 (5.2)	26.9 (5.5)	
**Anaemia (Hb < 11 g/dL), n (%)**				0.383¶
*n*	*116*	*58*	*58*	
	23 (19.8)	14 (24.1)	9 (15.5)	
**Vitamin D status**				**<0.001**
Serum 25(OH)D (nmol/L), mean (SD)	73.3 (28.1)	82.5 (28.8)	64.3 (24.5)	

Parity was matched by design (0, 1–3, or ≥4); no formal test of balance is reported.

SD: Standard deviation; BMI: Body mass index; Hb: Haemoglobin; 25(OH)D: 25-hydroxyvitamin D.

**Vitamin D (25(OH)D) was non-normally distributed; means (SD) are shown for descriptive purposes and comparability to prior studies.**

‡ P-value from paired t-test (continuous matched variables).

* P-value from the Stuart–Maxwell test for marginal homogeneity in matched pairs (polytomous categorical variables).

¶ P-value from McNemar's test (exact) for binary variables or Fisher's exact test for categorical variables with small cell counts.

Denominators (*n*) are provided for variables with missing data to indicate the actual sample size for that characteristic.

### Association between vitamin D deficiency and PE

Median maternal serum 25(OH)D concentration was 67.1 nmol/L (IQR 47.8–84.0) in PE cases vs. 77.6 nmol/L (IQR 62.4–99.7) in controls; means were 64.3 ± 24.5 vs 82.3 ± 28.6 nmol/L.

Using the IOM classification, deficiency (<30 nmol/L) was observed in 5 (8.5%) PE cases vs. 1 (1.7%) control and insufficiency (30–49 nmol/L) in 14 (23.7%) vs. 3 (5.1%) controls. Sufficient vitamin D levels (≥ 50 nmol/L) were present in 40 (67.8%) preeclampsia cases versus 55 (93.2%) controls. Table 2 presents the distribution of maternal serum 25(OH)D concentration by PE status.

**Table 2:** Association between vitamin D deficiency and preeclampsia (n = 118).

Vitamin D Status	Total (n = 118)	Controls (n = 59)	Preeclampsia Cases (n = 59)	*P*-value
**25(OH)D (nmol/L), median (IQR)**	69.9 (52.4–88.9)	79.4 (62.4–100.7)	67.1 (47.8–84.0)	0.001†
**Vitamin D category, n (%)**				**0.002‡**
Deficient (<30 nmol/L)	6 (5.1)	1 (1.7)	5 (8.5)	
Insufficient (30–49 nmol/L)	17 (14.4)	3 (5.1)	14 (23.7)	
Sufficient (≥50 nmol/L)	95 (80.5)	55 (93.2)	40 (67.8)	

a) *Serum 25(OH)D was right-skewed on normality testing. Median (IQR) values and non-parametric tests were therefore used for inference.*

b) † P-value derived from the Wilcoxon signed-rank test for matched pairs. Exact P = 0.0004;

c) ‡ P-value derived from McNemar's exact test for matched pairs.

**Fig 2:**
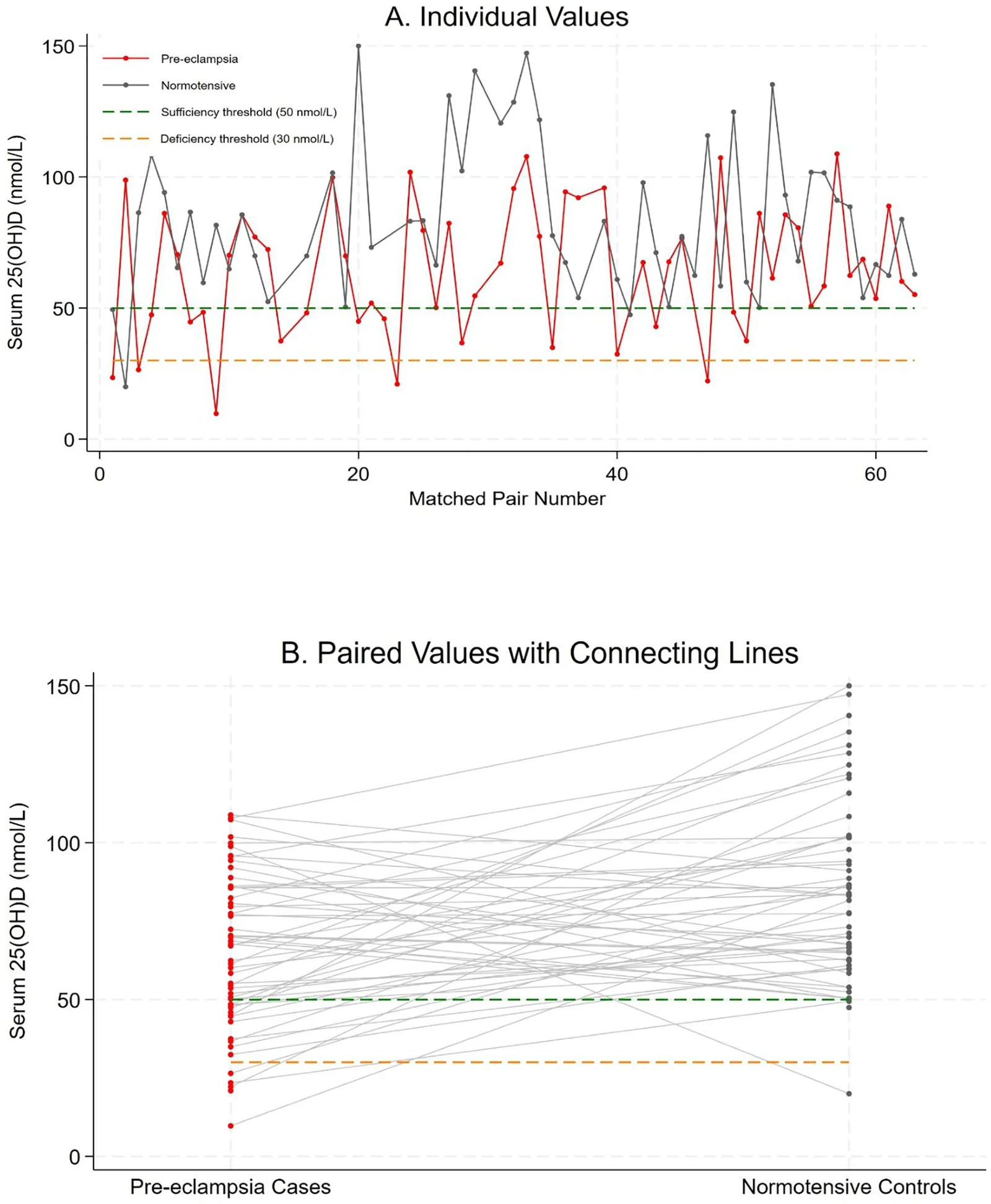
Individual and paired maternal serum 25(OH)D concentrations in cases and controls. In **panel A**, a slope graph displays serum 25(OH)D for each matched pair (red lines, PE cases; grey lines, controls). **Panel B** shows paired values connected by grey lines; green and orange dashed lines mark the 50 and 30 nmol/L thresholds, respectively.

Fig 2 presents individual and paired maternal serum 25(OH)D concentrations in cases and controls.

In Table 3, conditional logistic regression was used to examine the dose-response relationship between vitamin D status and PE. Women with deficiency (<30 nmol/L) had 5-fold odds of PE (OR 5.0; 95% CI: 0.58–42.8). However, due to the rarity of this deficiency category (5.1%), this estimate was imprecise. Using the < 50 nmol/L threshold, suboptimal vitamin D status was strongly associated with PE (OR 8.0; 95% CI: 1.84–34.78; *P* = 0.006). At the < 75 nmol/L threshold, the association was not statistically significant (OR 2.25; 0.98–5.17; *P =* 0.056), though the direction of effect was consistent with the primary findings. The 8-fold odds at the < 50 nmol/L threshold suggest this cutoff may be optimal for clinical risk stratification.

**Table 3:** Comparison across Vitamin D thresholds.

Threshold	Odds Ratio	95% CI	*P*-value	Preeclampsia, n (%)	Controls, n (%)
<30 nmol/L	5.0*	0.58 - 42.8	0.14	5 (8.5%)	1 (1.7%)
<50 nmol/L	8.0	1.84 - 34.78	0.006	19 (32.2%)	4 (6.8%)
<75 nmol/L	2.25	0.98–5.17	0.056	38 (64.4%)	28 (47.5%)

* The < 30 nmol/L estimate was derived from exact conditional logistic regression due to sparse data (6 events); all other estimates are from standard conditional logistic regression accounting for matched pairs (maternal age ± 3 years, gestational age ± 1 week, parity). No additional covariate adjustment was applied.

When examining maternal serum 25(OH)D as a continuous predictor, each 10 nmol/L decrease in vitamin D concentration was associated with a 29% increase in the odds of PE (aOR 1.29 per 10 nmol/L decrease; 95% CI 1.09–1.54; *P* = 0.004).

A stratified analysis by referral concordance within matched pairs showed that the maternal serum 25(OH)D concentration and PE association present in both referred and non-referred subgroups. Multivariable adjustment for BMI, referral status and anaemia did not materially alter the association between suboptimal vitamin D and PE (**S2 File).** However, the small sample size precluded formal exploration of non-linear dose-response relationships via restricted cubic splines.

## Discussion

Our study provides context on vitamin D status among a Kenyan obstetric study group. In a 2020 systematic review and meta-analysis of vitamin D deficiency in Africa, Mogire et al., reported pooled prevalence estimates of 34.2% for levels <50 nmol/L [13]. Notably, the study identified only 130 studies across 23 (of 54) countries on the continent. Pregnant women and new mothers exhibited higher deficiency prevalence (49%) compared with other adults (34%). Our findings mirror this vulnerability; 32.2% of PE cases had suboptimal vitamin D levels (<50 nmol/L), versus 6.8% of controls. This underscores suboptimal levels as a particularly important associated factor in this high-risk obstetric population. Its high burden challenges the assumption that abundant sunshine inherently precludes deficiency. Population-specific research remains important.

A significant association was seen between low maternal vitamin D levels and odds of PE. Women with suboptimal status (<50 nmol/L) exhibited 8-fold odds of PE compared to those with sufficient status. A linear dose-response relationship was observed with analysis showing a 29% increased odds of PE per 10 nmol/L decrement in 25(OH)D concentration.

Our results align with several other observational studies. In a United States (US) study, Bodnar et al., reported a 5-fold increased risk of PE development among nulliparous women in early pregnancy with 25(OH)D concentrations <37.5 nmol/L [20]. As well, Baker et al., reported a 5.4-fold risk of severe PE with 25(OH)D < 50 nmol/L [21]. In a study done in the DRC, the odds of PE were 3-fold in pregnant women with levels < 75 nmol/L [22].The study results show higher odds than these estimates, potentially reflecting the unique environmental and genetic context of participants. In a Chinese group of 605 pregnant women, 25(OH)D of <75 nmol/L was independently associated with PE (OR 10.24, p < 0.001) and demonstrated the highest individual diagnostic accuracy (Area Under the Curve 0.719) [23].

Kokkinari et al., summarised the molecular mechanisms by which vitamin D may protect against PE [24]. The key proposed mechanisms include suppression of pro-inflammatory cytokines (e.g., TNF-α, IL-6,), the upregulation of regulatory T cells to sustain maternal-fetal immune tolerance, and stimulation of angiogenic factors (VEGF, PIGF). These are key in spiral artery remodelling. Vitamin D-mediated suppression of RAAS also offers a plausible explanation for its antihypertensive effects. Evidence from oxidative stress biomarker studies also strengthens this inference [25,26]. Suboptimal vitamin D could potentially contribute to the pathophysiological cascade of PE.

An alternative explanation for the observed association is reverse causation. Because proteinuria is integral to the diagnosis of PE, glomerular injury may increase urinary loss of vitamin D-binding protein (VDBP) [27]. Reduced measured total 25(OH)D concentrations could be a consequence of established disease rather than as a predisposing factor.

Nevertheless, prospective studies that measured 25(OH)D in early or mid-gestation have consistently reported that lower vitamin D status precedes disease onset, supporting a temporal sequence in which deficiency may contribute to pathogenesis [21,28].

There could be potential value for antenatal screening of vitamin D status; particularly among women with risk of PE. A suboptimal threshold of 50 nmol/L shows a strong association in this data set. This aligns with recommendations from the Endocrine Society, defining deficiency as <50 nmol/L [4]. The dose-response relationship finding also supports consideration of vitamin D supplementation as a preventive intervention. One of the largest risk reductions reported was observed in an RCT performed in DRC by Kabuyanga et al. Monthly administration of 60,000 IU vitamin D from early pregnancy (with deficiency) resulted in a 64% reduction in PE risk (RR 0.36; 95% CI 0.19–0.69) [29]. A 2024 meta-analysis of 33 RCTs similarly reported a 45% reduction [9]. However, a 2024 Cochrane review of 10 studies involving 2313 pregnant women found only very low-certainty evidence for vitamin D supplementation on PE, gestational diabetes, preterm birth, or nephritic syndrome [30]. Currently, the World Health Organization (WHO) *Recommendations on Antenatal Care for a Positive Pregnancy Experience* does not recommend universal vitamin D supplementation in pregnancy [31].

Strengths and limitations: The matched nature of the study design reduced confounding, and conditional logistic regression appropriately accounted for it.

The outcomes also add to the data necessary to evaluate whether vitamin D assessment warrants integration into PE care in Kenya. This represents an incremental contribution to literature.

Case-control design disqualifies any establishment of causality. Vitamin D was measured at a single time point (≥28 weeks). Determination of whether deficiency preceded PE onset could not be determined. The observed association should therefore be viewed as hypothesis-generating rather than evidence of causality. Among the 118 matched participants, 16.9% were enrolled at <32 weeks and 38.1% at 32–36 weeks’ gestation; moreover, all six participants excluded for lack of a match were <37 weeks. Our findings therefore apply mainly to PE presenting from 28 weeks onwards, are least informative for early-onset PE < 32 weeks, and should be extrapolated to that subgroup with caution.

The relatively small sample size resulted in wide confidence intervals for some estimates, particularly for the severe deficiency category (<30 nmol/L). Self-reported data on vitamin D supplementation, prenatal multivitamin use, daily outdoor hours, and sunscreen use were collected. These variables were however not included in the primary conditional logistic regression model. Vitamin D supplementation was infrequent with 4 of 124 enrolled participants reporting any use. All were at less than 400 IU per day. Supplementation and multivitamin use could therefore not be modelled reliably as covariates. Any residual confounding from supplementation was likely minimal, as use was rare, low-dose, and similarly distributed between cases and controls. Skin pigmentation intensity was not quantified. It remains a genuine unmeasured confounder. Seasonality was mitigated by recruiting across both dry and wet seasons. It was not included as an analytical covariate.

Lastly, the single-centre design may limit generalisability to community settings, though the diverse catchment population enhances external validity.

## Conclusion

In this study, suboptimal maternal serum vitamin D status demonstrated a significant association with PE. There is potential value of antenatal screening for vitamin D status. Findings support further research into vitamin D supplementation as a preventive intervention for PE.

## Supporting information

S1 File STROBE checklist. (DOCX)

S2 File Supplementary analyses, with tables S1–S4. (DOCX)

S3 File De-identified dataset. (XLSX)
