# Supplementary material for: Maternal serum vitamin D levels and preeclampsia among Kenyan pregnant women: A matched case-control study
Source: PLoS One. 2026 Sep 22;21(9):e0358781. doi: 10.1371/journal.pone.0358781 (PMC13596797; doi:10.1371/journal.pone.0358781)
Supplement: Supplementary file 1 [file pone.0358781.s001.docx]

STROBE Statement—Checklist of items that should be included in reports of ***case-control studies***

|  | **Item**  **No** | **Recommendation** | **Location in Manuscript** |
| --- | --- | --- | --- |
| **Title and abstract** | 1 | (*a*) Indicate the study’s design with a commonly used term in the title or the abstract | "Title: 'a matched case-control study'; Abstract, Methods: 'A matched case-control study'" |
|  |  | (*b*) Provide in the abstract an informative and balanced summary of what was done and what was found | Page 1: Structured abstract (Objective/Methods/Results/Conclusion) |
| **Introduction** |  |  |  |
| Background/rationale | 2 | Explain the scientific background and rationale for the investigation being reported | Page 1 (Introduction): Rationale for Vitamin D in Kenyan population |
| Objectives | 3 | State specific objectives, including any prespecified hypotheses | Page 1 (end of Introduction): Two specific objectives listed |
| **Methods** |  |  |  |
| Study design | 4 | Present key elements of study  design early in the paper | Title, Abstract (line 1), and Methods (page 2, first  paragraph): “matched case-control study” |
| Setting | 5 | Describe the setting, locations, and relevant dates, including periods of recruitment, exposure,  follow-up, and data collection | Page 2: “Moi Teaching and Referral Hospital (MTRH)... Eldoret, Kenya... 12,000 deliveries annually... Aug 1st 2025 to Jan 31st 2026” “Vitamin D measured at enrollment ≥28 weeks” |
| Participants | 6 | (*a*) Give the eligibility criteria, and the sources and methods of case ascertainment and control selection. Give the rationale for the choice of cases and controls | Page 4: Eligibility criteria listed. Case ascertainment via trained research assistants reviewing daily ward admission registers using facility protocols. Controls selected concurrently from same antenatal/labour wards. Rationale for hospital-based controls stated - comparable healthcare access. |
|  |  | (*b*) For matched studies, give matching criteria and the number of controls per case | Page 4 and 5: Matching criteria specified (maternal age ±3 years, gestational age ±1 week, parity categorized as 0, 1, or ≥2). Number of controls per case stated as 1:1 (59 matched pairs). Matching accounted for in analysis via conditional logistic  regression (mentioned in Statistical Analysis). |
| Variables | 7 | Clearly define all outcomes, exposures, predictors, potential confounders, and effect modifiers. Give diagnostic criteria, if applicable | Page 4,5: preeclampsia defined using International Society for Study of Hypertension in Pregnancy criteria. Vitamin D categorized using Institute of Medicine thresholds and Endocrine Society figures. |

Data sources/ measurement

8*	For each variable of interest, give sources of data and

Page 5: Data sources specified: 5 mL venous blood collected in serum separator tubes, analysed at Cerba

|  |  | details of methods of assessment (measurement). Describe comparability of assessment methods if there is  more than one group | Lancet laboratory (ISO15189 certified). Covariates from structured interviewer-administered questionnaires and medical files. Comparability addressed by blinding laboratory technicians to  case/control status. |
| --- | --- | --- | --- |
| Bias | 9 | Describe any efforts to address potential sources of bias | Page 5: exclusion of pre-existing conditions affecting vitamin D metabolism, blinding of laboratory technicians processing vitamin D assays, matched study design. |
| Study size | 10 | Explain how the study size was arrived at | Page 3: based on prior African data, 59 case-control pairs provided 80% power to detect a 25-percentage-point difference in deficiency prevalence (two-proportion comparison, α=0.05). |
| Quantitative variables | 11 | Explain how quantitative variables were handled in the analyses. If applicable, describe which groupings were chosen and why | Page 5: Statistical analysis: Quantitative variables (serum 25(OH)D) handled as continuous (dose-response) and categorical (IOM and Endocrine Society figures). Continuous variables summarized as means (SD) or medians (IQR) based on Kolmogorov-Smirnov normality testing. |
| Statistical methods | 12 | (*a*) Describe all statistical methods, including those used to control for confounding | Page 5: Conditional logistic regression used to account for matched design (confounding control). Paired t-tests, Wilcoxon signed-rank tests, and McNemar's/Fisher's exact tests used for matched comparisons. |
|  |  | (*b*) Describe any methods used to examine subgroups and interactions | N/A |
|  |  | (*c*) Explain how missing data were addressed | Page 5, table 1: “Missing data were handled by complete case analysis.” Denominators provided in Table 1 for variables with missing data (e.g., BMI  n=114, Anemia n=116). |
|  |  | (*d*) If applicable, explain how matching of cases and controls was addressed | Page 5: Matching addressed via conditional logistic regression for main association. Baseline comparisons used paired tests (paired t-tests, Wilcoxon signed-rank, McNemar's) accounting for 1:1 matched pairs. |
|  |  | (*e*) Describe any sensitivity analyses | S2 File: multivariable conditional logistic regression adjusting for BMI, referral status, and anemia |
| **Results** |  |  |  |
| Participants | 13* | (a) Report numbers of individuals at each stage of study—eg numbers potentially eligible, examined for eligibility, confirmed eligible, included in the study, completing follow-up, and analysed | 3 parathyroid conditions, 2 chronic kidney disease)  → 6 declined participation → 124 enrolled → 6 excluded post-hoc (matching criteria audit) → 118 analyzed (59 matched pairs). |

(b) Give reasons for non-participation at each stage

|  |  | (c) Consider use of a flow diagram | Figure 1. Supplementary file. |
| --- | --- | --- | --- |
| Descriptive data | 14* | (a) Give characteristics of study participants (eg demographic, clinical, social) and information on exposures and potential confounders | Table 1; Results → Participant characteristics and vitamin D status |
|  |  | (b) Indicate number of participants with missing data for each variable of interest | Missing data indicated in footnotes of Table 1: BMI missing for 4 participants (n=114/118), anemia data missing for 2 participants (n=116/118). |
| Outcome data | 15* | Report numbers in each exposure category, or summary measures of  exposure | Table 2; Results → Association between vitamin D deficiency and PE. |
| Main results | 16 | (*a*) Give unadjusted estimates and, if applicable, confounder-adjusted estimates and their precision  (eg, 95% confidence interval). Make clear which confounders were adjusted for  and why they were included | Table 3; Results → Association between vitamin D deficiency and PE; Supplementary 2 (Table 5).  Unadjusted estimates provided. Confounder-adjusted estimates (BMI, referral status, anemia) reported in Supplementary Table 5. Main manuscript presents only crude estimates. |
|  |  | (*b*) Report category boundaries when continuous variables were categorized | Table 2 and Table 3. Category boundaries explicitly defined: deficient (<30 nmol/L), insufficient (30–49 nmol/L), sufficient (≥50 nmol/L), and suboptimal (<50 nmol/L) per IOM and Endocrine Society guidelines. Table 3 additionally reports <75 nmol/L threshold. Dose-response analyses specify 10 nmol/L decrements. |
|  |  | (*c*) If relevant, consider translating estimates of relative risk into absolute risk  for a meaningful time period | N/A |

Page 7: Reasons for decline included anxiety about needles and excessive blood loss (3) and perceived lack of direct benefit (2). One woman in labour declined due to competing clinical priorities.

| Other analyses | 17 | Report other analyses done—eg analyses of subgroups and interactions, and sensitivity analyses | Supplementary file 2. |
| --- | --- | --- | --- |
| **Discussion** |  |  |  |
| Key results | 18 | Summarise key results with reference to study objectives | Page 7 paragraph 1:  suboptimal vitamin D (<50 nmol/L) (32.2% vs 6.8%),  8-fold increased odds of PE(OR 8.0; 95% CI: 1.84–  34.78), and dose-response relationship (29% increase per 10 nmol/L decrement). |
| Limitations | 19 | Discuss limitations of the study, taking into account sources of potential bias or imprecision. Discuss both direction and magnitude of any potential bias | Page 13; Strengths and limitations (1) Case-control design precludes causality; (2) Single time-point measurement (3) Small sample size and (4) Single-center design |
| Interpretation | 20 | Give a cautious overall interpretation of results considering objectives, limitations, multiplicity of analyses, results from similar studies, and other  relevant evidence | Page 13; Discussion (paragraphs 2–4) Cautious interpretation provided: results contextualized within existing literature. |
| Generalisability | 21 | Discuss the generalisability (external validity) of the study results | Page 13; Strengths and limitations. : single-center tertiary hospital design limits applicability to community settings; however, diverse referral catchment enhances external validity for similar high-altitude, multi-ethnic populations in East Africa. |
| **Other information** | | | |
| Funding | 22 | Give the source of funding and the role of the funders for the present study and, if applicable, for the original study on which the present article is based | Page 14; Grant Information section (following Conclusions). Funding source declared: Bill & Melinda Gates Foundation (grant ID: INV-033705) awarded to first author as part of Supporting Women in Science program at Aga Khan University-Institute  for Global Health and Development. |

*Give information separately for cases and controls.

**Note:** An Explanation and Elaboration article discusses each checklist item and gives methodological background and published examples of transparent reporting. The STROBE checklist is best used in conjunction with this article (freely available on the Web sites of PLoS Medicine at <http://www.plosmedicine.org/>, Annals of Internal Medicine at <http://www.annals.org/>, and Epidemiology at <http://www.epidem.com/>). Information on the STROBE Initiative is available at [http://www.strobe-statement.org](http://www.strobe-statement.org/).
