# Supplementary material for: Maternal serum vitamin D levels and preeclampsia among Kenyan pregnant women: A matched case-control study
Source: PLoS One. 2026 Sep 22;21(9):e0358781. doi: 10.1371/journal.pone.0358781 (PMC13596797; doi:10.1371/journal.pone.0358781)
Supplement: Supplementary file 2 [file pone.0358781.s002.docx]

# Table S1. Collinearity diagnostics for conditional logistic regression models

| **Variable** | **Model 1** | **Model 2** | **Model 3** | **Model 4** | **Correlation with Vitamin D*** |
| --- | --- | --- | --- | --- | --- |
| Suboptimal Vitamin D (<50 nmol/L) | 1.12 | 1.12 | 1.12 | — | Reference |
| Vitamin D continuous (per 10 nmol/L) | — | — | — | 1.12 | r = -0.64 |
| BMI (kg/m²) | 1.01 | 1.01 | 1.01 | 1.01 | ≈ 0.00‡ |
| Referred status | — | 1.06 | 1.06 | 1.06 | r = 0.28 |
| Anemia (Hb <11 g/dL) | — | — | 1.06 | — | r = 0.20 |
| Maximum VIF | 1.00 | 1.05 | 1.07 | 1.07 | All <1.2 |
| Collinearity assessment | None | None | None | None | No concerns |

** Pearson correlation coefficient with suboptimal vitamin D indicator*

*Model 1 : Suboptimal Vitamin D + BMI*

*Model 2: Suboptimal Vitamin D + BMI + Referred status Model 3: Suboptimal Vitamin D + BMI + Referred + Anemia*

*Model 4: Continuous Vitamin D (per 10 nmol/L) + BMI + Referred*

Variance Inflation Factors (VIF) were approximated via OLS regression on the complete-case subset. All values were <1.2 (range: 1.01–1.12). Matching variables were not included as covariates in the models.

The association between suboptimal vitamin D and PE had the strongest effect after BMI adjustment (aOR 14.30). This suggests that BMI acts as a negative confounder: higher BMI reduces circulating 25(OH)D levels through volumetric dilution and sequestration in adipose tissue, while also independently increasing risk of PE through metabolic and inflammatory pathways. The weakening of effect after referral status adjustment indicates that women referred from peripheral facilities, had both lower vitamin D levels and higher baseline PE risk, creating confounding by indication **(Table S2)**.

# Table S2. Conditional logistic regression

| **Model** | **Vitamin D levels** | **Adjustment Variables** | **n** | **aOR (95% CI)** | **P-value** |
| --- | --- | --- | --- | --- | --- |
|  | <50 nmol/L | Matching |  | **8.00 (1.84–** |  |
| **Crude** |  | only* | 116 | **34.78)** | **0.006** |
|  |  |  |  | **14.30** |  |
| **Model 1** | <50 nmol/L | BMI | 108 | **(1.87–**  **109.57)** | **0.010** |
|  |  | BMI + |  |  |  |
| **Model 2** | <50 nmol/L | Referred  status | 108 | **8.34 (1.02–**  **68.44)** | **0.048** |
|  |  | BMI + |  | **13.45** |  |
| **Model 3** | <50 nmol/L | Referred +  Anemia | 106 | **(1.35–**  **134.13)** | **0.027** |
|  | Per 10 |  |  |  |  |
| **Model 4** | nmol/L | Matching |  | **1.30 (1.09–** |  |
| **(Crude)** | decrease | only* | 116 | **1.54)** | **0.003** |
|  | Per 10 |  |  |  |  |
| **Model 4** | nmol/L | BMI + |  | 1.17 (0.96– |  |
| **(Adjusted)** | decrease | Referred | 108 | 1.43) | 0.129 |

* *Matching variables (maternal age, gestational age, parity) controlled by design via conditional logistic regression.*

*aOR, adjusted odds ratio; CI, confidence interval.*

Adjusted models used complete-case analysis. Matched pairs with missing covariate data in either member were excluded. The dataset yielded n = 108 for BMI-adjusted models and n = 106 for the fully adjusted model. This reflected four pairs with missing BMI and one additional pair with missing haemoglobin. Stata additionally omitted one concordant pair per model due to all-positive or all-negative outcomes within the stratum.

Excluded participants were 6 enrolled individuals (3 PE cases, 3 normotensive controls) who could not be matched according to the protocol-specified criteria. Fisher's exact test was used for case/control balance; Wilcoxon rank-sum test for continuous variables. The excluded group had significantly lower gestational age than included participants (median 31.5 vs. 36 weeks; P = 0.011). This was a reflection of the difficulty of matching very preterm or early-term PE cases to controls. No statistically significant differences were observed in age, vitamin D status, BMI, or case-control balance **(Table S3).**

**Table S3.** Comparison of excluded and included participants

| **Characteristic** | **Included (*n* = 118)** | **Excluded (*n* = 6)** | ***P*-value** |
| --- | --- | --- | --- |
| **Case/Control, n** | 59 / 59 | 3 / 3 | 1.000 |
| **Maternal age (years), mean (SD)** | 28.9 (6.2) | 28.2 (4.0) | 0.784 |
| **Gestational age (weeks), median (IQR)** | 36 (33–38) | 31.5 (30–33) | **0.011** |
| **BMI in early pregnancy (kg/m²), median (IQR)** | 25.8 (22.5–30.1) | 29.4 (24.8–33.6) | 0.168 |
| **Serum 25(OH)D (nmol/L), median (IQR)** | 69.9 (52.4–88.9) | 56.5 (39.9–119.8) | 0.604 |
| **Level of education, n (%)** |  |  |  |
| No formal schooling | 3 (2.5) | 1 (16.7) | — |
| Primary completed | 22 (18.6) | 0 (0.0) |  |
| Secondary completed | 42 (35.6) | 2 (33.3) |  |
| College/University completed | 51 (43.2) | 3 (50.0) |  |
| **Work status, n (%)** |  |  |  |
| Homemaker | 9 (7.6) | 2 (33.3) | — |
| Non-government employee | 12 (10.2) | 1 (16.7) |  |
| Self-employed | 20 (16.9) | 0 (0.0) |  |
| Student | 7 (5.9) | 0 (0.0) |  |
| Unemployed (able to work) | 63 (53.4) | 1 (16.7) |  |
| Unemployed (unable to work) | 7 (5.9) | 2 (33.3) |  |
| **Marital status, n (%)** |  |  |  |
| Currently married | 97 (82.2) | 3 (50.0) | — |
| Never married | 17 (14.4) | 3 (50.0) |  |
| Other | 4 (3.4) | 0 (0.0) |  |
| **Referred from peripheral facility, n (%)** | 69 (58.5) | 2 (33.3) | — |

The following stratified analysis is presented as a sensitivity analysis to examine whether the association is driven exclusively by the referred subgroup. The association is present in both referred and non-referred concordant pairs, and in discordant pairs where the case was referred, supporting the robustness of the primary finding **(Table S4).**

**Table S4.** Stratified analysis of the association between maternal serum 25(OH)D and pre-eclampsia by referral concordance within matched pairs

| **Referral concordance status** | **Matched pairs, n** | **Pre-eclampsia cases** |
| --- | --- | --- |
|  |  | Serum 25(OH)D, median (IQR), nmol/L |
| **Both referred** | 20 | 60.8 (42.1–79.0) |
| **Both non-referred** | 10 | 79.6 (66.2–100.3) |
| **Discordant: case referred, control not referred** | 26 | 55.4 (36.2–81.1) |
| **Discordant: control referred, case not referred** | 3 | 77.1 (72.4–107.8) |
|  | | |
| **Vitamin D category by referral concordance status** | | |
| **Referral concordance status** | **Matched pairs, n** | **Cases with suboptimal 25(OH)D (<50 nmol/L), n (%)** |
| **Both referred** | 20 | 6 (30.0) |
| **Both non-referred** | 10 | 1 (10.0) |
| **Discordant: case referred, control not referred** | 26 | 10 (38.5) |
| **Discordant: control referred, case not referred** | 3 | 2 (66.7) |

**Footnotes**

a) *Referral concordance was defined at the matched-pair level. "Both referred" indicates that the PE case and matched normotensive control were both referred from a peripheral health facility to Moi Teaching and Referral Hospital (MTRH). "Both non-referred" indicates that both members of the pair presented directly to MTRH. "Discordant" pairs indicate that one member was referred and the other presented directly.*

c) *The Wilcoxon signed-rank test was applied to concordant pairs (both referred; both non-referred) where the matched structure is preserved.*

d) *McNemar's exact test for suboptimal vitamin D (<50 nmol/L) was not performed within individual strata because of the small sample sizes. The categorical data are presented descriptively for transparency.*
