# Supplementary material for: Maternal serum vitamin D levels and preeclampsia among Kenyan pregnant women: A matched case-control study
Source: PLoS One. 2026 Sep 22;21(9):e0358781. doi: 10.1371/journal.pone.0358781 (PMC13596797; doi:10.1371/journal.pone.0358781)
Supplement: Supplementary file 4 [file pone.0358781.s006.docx]

**Response to Reviewers**

**Manuscript number: PONE-D-26-14449**

**Manuscript title:** Maternal Serum Vitamin D Levels and Preeclampsia among Kenyan Pregnant Women: A matched case-control study

**Corresponding author:** Kimbley Asaso Omwodo

________________________________________

We thank the Editor and both reviewers for their continued careful assessment of our manuscript. We are grateful to Reviewer #1 for confirming that the previous comments have been addressed. We have addressed each of Reviewer #2’s remaining points below and have made the corresponding changes in the revised manuscript, supporting information files, and submission metadata.

**Reviewer #1**

**Comment: All comments have been addressed; the paper can now be published.**

**Response:** We thank Reviewer #1 for their time and constructive feedback throughout the review process. We are pleased that the revisions are satisfactory.

**Reviewer #2**

We thank Reviewer #2 for acknowledging the improvements, and for identifying the remaining issues. We have addressed each point as follows.

**Major Comment 1**

**Comment: Correct the remaining arithmetic inconsistency in Table 2 (Mandatory). The manuscript still contains one unresolved numerical inconsistency in Table 2. The control group vitamin D categories currently report: Deficient = 1, Insufficient = 3, Sufficient = 54. These values sum to 58, whereas the control sample size is reported as 59. Consequently, the overall total also sums to 117 instead of 118. Based on the reported suboptimal vitamin D counts elsewhere in the manuscript, the internally consistent values should be: - Controls: 55 participants (93.2%) with sufficient vitamin D. - Overall: 95 participants (80.5%) with sufficient vitamin D. Please correct Table 2 and the corresponding sentence in the Results section and verify that all percentages are recalculated using the correct denominators.**

**Response:** We agree and apologise for the residual inconsistency. Table 2 has been corrected so that the control-group categories now read: deficient = 1 (1.7%), insufficient = 3 (5.1%), and sufficient = 55 (93.2%), summing to 59 controls. The overall row now reads: deficient = 6 (5.1%), insufficient = 17 (14.4%), and sufficient = 95 (80.5%), summing to 118 participants. The corresponding Results sentence has been updated to state that sufficient vitamin D levels (>=50 nmol/L) were present in 40 (67.8%) preeclampsia cases versus 55 (93.2%) controls. All percentages in Table 2 were recalculated using the corrected denominators.

**Major Comment 2**

**Comment: Verify the originality argument after reference renumbering.**

**Response**: We re-verified all in-text citations against the final, renumbered reference list. The novelty statement (final paragraph of the Introduction; first paragraph of the Discussion) is appropriately cautious, describing the study as 'context-specific' and an 'incremental contribution', and is supported by the cited African systematic review (Mogire et al., reference 13). This check identified one mis-citation introduced by the renumbering: the Endocrine Society guideline is now cited as reference 4 (previously reference 5).

**Minor Comment 3**

**Comment: Ensure the Ethics Statement in the submission system matches the manuscript text by using MTRH and NACOSTI consistently.**

**Response:** The Ethics Statement in the submission metadata has been updated to use 'MTRH' and 'NACOSTI' consistently, matching the manuscript text (IREC approval 0005019; NACOSTI licence NACOSTI/P/25/417821).

**Minor Comment 4**

**Comment: Reconcile the Supporting Information file format.**

**Response:** We have harmonised the Supporting Information: S1 File (completed STROBE checklist, PDF), S2 File (supplementary analyses, with tables relabelled S1–S5 Table), and S3 File (de-identified dataset, XLSX). The Supporting Information legend and all in-text citations have been updated accordingly.

**Minor Comment 5**

**Comment: The manuscript states that vitamin D supplementation or multivitamin use exhibited "high collinearity with matching factors." This assertion is not supported by any statistical evidence presented in the manuscript. Unless supporting diagnostics are provided, I recommend replacing this statement with a simpler explanation indicating that supplementation was too infrequent to be modeled reliably. Also clarify that the supplementation denominator refers to the 124 enrolled participants, whereas the analytical sample consisted of 118 matched participants.**

**Response:** We agree and have removed the unsupported collinearity statement. The revised text now states that vitamin D supplementation was too infrequent to model reliably: *‘Vitamin D supplementation was infrequent with 4 of 124 enrolled participants reporting any use. All were at less than 400 IU per day. Supplementation and multivitamin use could therefore not be modelled reliably as covariates. Any residual confounding from supplementation was likely minimal, as use was rare, low-dose, and similarly distributed between cases and controls.’*

**Minor Comment 6**

**Comment:**  **Ensure consistency in the multivariable adjustment description. The Results section states that adjustment for BMI and anemia did not materially alter the association. However, the supplementary analyses indicate that referral status was also included in the adjusted models. Please revise the Results sentence so that it consistently states adjustment for BMI, referral status, and anemia.**

**Response:** The Results sentence now consistently states that multivariable adjustment for BMI, referral status, and anemia did not materially alter the association between suboptimal vitamin D and preeclampsia. This wording matches the adjusted conditional logistic regression models presented in S2 File. We also clarified that complete-case adjusted models used smaller denominators because of missing BMI and haemoglobin data.

**Minor Comment 7**

**Comment: The limitation currently states that the findings primarily apply to preeclampsia presenting at or beyond 28 weeks' gestation. However, based on the authors' own data, a notable proportion of participants were enrolled before 32 weeks and between 32–36 weeks.**

**Including this gestational-age distribution in the limitations section would improve transparency regarding the applicability of the findings across the preeclampsia spectrum.**

**Response:** We have expanded the limitation to report the gestational-age distribution of the analytic cohort. *‘’Among the 118 matched participants, 16.9% were enrolled at <32 weeks and 38.1% at 32–36 weeks' gestation; moreover, all six participants excluded for lack of a match were <37 weeks. Our findings therefore apply mainly to PE presenting from 28 weeks onwards, are least informative for early-onset PE <32 weeks, and should be extrapolated to that subgroup with caution.’’*

**Minor Comment 8**

**Comment: Clarify whether the cited WHO guidance is regional or global.**

**Response:** We agree the citation was regional guidance. We have replaced it with the global WHO antenatal care recommendations (WHO, 2016), which do not recommend universal vitamin D supplementation in pregnancy to prevent preeclampsia; the text now accurately reflects the scope of the cited guidance.

**Final cross-check requested by Reviewer #2**

**Response: Before resubmission, we performed the requested final cross-check of: sample sizes and denominators throughout the manuscript; frequencies and percentages across all tables; table, figure, and supporting-information numbering; figure captions and legends; in-text citations after reference renumbering; and consistency among the manuscript, supplementary materials, and submission metadata.**

**Corrections arising from this cross-check are reflected in the revised manuscript and supporting files.**

We hope that these revisions adequately address the remaining concerns and that the manuscript is now suitable for publication.

Sincerely,

The Authors
