# Supplementary material for: Maternal serum vitamin D levels and preeclampsia among Kenyan pregnant women: A matched case-control study
Source: PLoS One. 2026 Sep 22;21(9):e0358781. doi: 10.1371/journal.pone.0358781 (PMC13596797; doi:10.1371/journal.pone.0358781)
Supplement: Peer review history

# Peer review: Decision Letter 0

**DOI:** 10.1371/journal.pone.0358781.r001

**Authors:** Maria Magnus

23 Jul 2026

Dear Dr. Asaso Omwodo,

Thank you for submitting your manuscript to PLOS ONE. After careful consideration, we feel that it has merit but does not fully meet PLOS ONE’s publication criteria as it currently stands. Therefore, we invite you to submit a revised version of the manuscript that addresses the points raised during the review process.

Please submit your revised manuscript by Sep 06 2026 11:59PM. If you will need more time than this to complete your revisions, please reply to this message or contact the journal office at plosone@plos.org. When you're ready to submit your revision, log on to https://www.editorialmanager.com/pone/ and select the 'Submissions Needing Revision' folder to locate your manuscript file.

- A letter that responds to each point raised by the academic editor and reviewer(s). You should upload this letter as a separate file labeled 'Response to Reviewers'.
- A marked-up copy of your manuscript that highlights changes made to the original version. You should upload this as a separate file labeled 'Revised Manuscript with Track Changes'.
- An unmarked version of your revised paper without tracked changes. You should upload this as a separate file labeled 'Manuscript'.

If you would like to make changes to your financial disclosure, please include your updated statement in your cover letter. Guidelines for resubmitting your figure files are available below the reviewer comments at the end of this letter.

If applicable, we recommend that you deposit your laboratory protocols in protocols.io to enhance the reproducibility of your results. Protocols.io assigns your protocol its own identifier (DOI) so that it can be cited independently in the future. For instructions see: https://journals.plos.org/plosone/s/submission-guidelines#loc-laboratory-protocols. Additionally, PLOS ONE offers an option for publishing peer-reviewed Lab Protocol articles, which describe protocols hosted on protocols.io. Read more information on sharing protocols at https://plos.org/protocols?utm_medium=editorial-email&utm_source=authorletters&utm_campaign=protocols.

As the corresponding author, your ORCID iD is verified in the submission system and will appear in the published article. PLOS supports the use of ORCID, and we encourage all coauthors to register for an ORCID iD and use it as well. Please encourage your coauthors to verify their ORCID iD within the submission system before final acceptance, as unverified ORCID iDs will not appear in the published article. *Only*  the individual author can complete the verification step; PLOS staff *cannot*  verify ORCID iDs on behalf of authors.

We look forward to receiving your revised manuscript.

Kind regards,

Maria Christine Magnus, PhD

Academic Editor

PLOS One

Journal requirements:

When submitting your revision, we need you to address these additional requirements.

1. Please ensure that your manuscript meets PLOS ONE's style requirements, including those for file naming. The PLOS ONE style templates can be found at

https://journals.plos.org/plosone/s/file?id=wjVg/PLOSOne_formatting_sample_main_body.pdf and

https://journals.plos.org/plosone/s/file?id=ba62/PLOSOne_formatting_sample_title_authors_affiliations.pdf

2. Please amend either the abstract on the online submission form (via Edit Submission) or the abstract in the manuscript so that they are identical.

3. We note that there is identifying data in the Supporting Information file <Supplementary 3. Deidentified dataset.xlsx>. Due to the inclusion of these potentially identifying data, we have removed this file from your file inventory. Prior to sharing human research participant data, authors should consult with an ethics committee to ensure data are shared in accordance with participant consent and all applicable local laws.

Data sharing should never compromise participant privacy. It is therefore not appropriate to publicly share personally identifiable data on human research participants. The following are examples of data that should not be shared:

-Name, initials, physical address

-Ages more specific than whole numbers

-Internet protocol (IP) address

-Specific dates (birth dates, death dates, examination dates, etc.)

-Contact information such as phone number or email address

-Location data

-ID numbers that seem specific (long numbers, include initials, titled “Hospital ID”) rather than random (small numbers in numerical order)

Data that are not directly identifying may also be inappropriate to share, as in combination they can become identifying. For example, data collected from a small group of participants, vulnerable populations, or private groups should not be shared if they involve indirect identifiers (such as sex, ethnicity, location, etc.) that may risk the identification of study participants.

Additional guidance on preparing raw data for publication can be found in our Data Policy (https://journals.plos.org/plosone/s/data-availability#loc-human-research-participant-data-and-other-sensitive-data) and in the following article: http://www.bmj.com/content/340/bmj.c181.long.

Please remove or anonymize all personal information, ensure that the data shared are in accordance with participant consent, and re-upload a fully anonymized data set. Please note that spreadsheet columns with personal information must be removed and not hidden as all hidden columns will appear in the published file.

4. Please include captions for your Supporting Information files at the end of your manuscript, and update any in-text citations to match accordingly. Please see our Supporting Information guidelines for more information: http://journals.plos.org/plosone/s/supporting-information.

If the reviewer comments include a recommendation to cite specific previously published works, please review and evaluate these publications to determine whether they are relevant and should be cited. There is no requirement to cite these works unless the editor has indicated otherwise.

Additional Editor Comments:

You paper has undergone peer review. Several issues have been highlighted. If you manuscript is to be further considered for publication, you need to carefully adress all comments provided by the reviewers. In particular, you need to carefully assess the manuscript for any inconsistencies as pointed out.

[Note: HTML markup is below. Please do not edit.]

Reviewers' comments:

Reviewer's Responses to Questions

**Comments to the Author**

1. Is the manuscript technically sound, and do the data support the conclusions?

Reviewer #1: Yes

Reviewer #2: Partly

**********

2. Has the statistical analysis been performed appropriately and rigorously?

Reviewer #1: N/A

Reviewer #2: No

**********

3. Have the authors made all data underlying the findings in their manuscript fully available?

The PLOS Data policy

Reviewer #1: Yes

Reviewer #2: Yes

**********

4. Is the manuscript presented in an intelligible fashion and written in standard English?

Reviewer #1: Yes

Reviewer #2: Yes

**********

Reviewer #1: This is an interesting study. However, considering that the findings and the conclusions of this study are almost similar to those of Reference 23, some more comments are necessary in your manuscript about the "originality" of your findings.

Reviewer #2: Thank you for the opportunity to review this manuscript entitled "Maternal Serum Vitamin D Levels and Pre-eclampsia among Kenyan Pregnant Women: A Matched Case-Control Study." This study addresses an important clinical and public health question in an area where high-quality African data remain relatively limited. The manuscript has several notable strengths, including prospective registration (PACTR), availability of a previously published protocol, use of a DEQAS-participating accredited laboratory for vitamin D assays, an appropriate matched case-control design, STROBE-guided reporting, and the provision of a de-identified dataset, which enhances transparency and reproducibility. Overall, the work has the potential to make a valuable contribution to the literature. However, several methodological, statistical, reporting, and interpretative issues should be addressed before the manuscript is suitable for publication. Most of these concerns appear remediable through clarification, correction of inconsistencies, and additional discussion.

Major Comments

1. Internal numerical inconsistencies require correction

Several discrepancies were identified between the Abstract, Results section, and Table 2. These should be reconciled against the original dataset before publication.

• The reported interquartile ranges (IQRs) for maternal serum vitamin D differ across the Abstract, Results narrative, and Table 2.

• The number and percentage of controls classified as vitamin D sufficient are inconsistent. The Results text reports 54 (91.5%), whereas Table 2 reports 55 (93.2%). Internal arithmetic supports the Table 2 values.

• The reported prevalence of suboptimal vitamin D among controls is inconsistently presented as 6.8% and 6.9%. Please standardize rounding throughout the manuscript.

Because these discrepancies affect confidence in the reported findings, all numerical values should be carefully verified and made fully consistent across the manuscript.

2. Clarify the statistical analyses used for matched data

The manuscript describes a matched case-control study; however, several statistical procedures appear inconsistent with the matched design.

• The Methods and Table 2 indicate use of the Mann–Whitney U (Wilcoxon rank-sum) test for skewed continuous variables. Since observations are matched, the appropriate non-parametric paired alternative would generally be the Wilcoxon signed-rank test. If the rank-sum test was intentionally used, please justify this approach or consider reanalysis using methods appropriate for matched data.

• Similarly, Table 1 reports baseline categorical comparisons using conditional logistic regression likelihood-ratio tests. This approach is unusual for descriptive baseline comparisons in matched studies. Please clarify the rationale or consider using conventional matched categorical tests where appropriate.

3. Remove the post hoc power calculation

The reported post hoc (achieved) power calculated from the observed effect size does not provide meaningful additional information and is generally discouraged because it is mathematically related to the observed p-value. Instead, I recommend removing this analysis and emphasizing confidence intervals and the precision of the estimates.

4. Clarify exclusion of six enrolled participants

The manuscript states that six participants were excluded after a post hoc audit of the protocol-specified matching criteria. Additional transparency is needed.

Please clarify:

• precisely how "unmatched" was defined,

• why matching was not resolved during recruitment,

• whether exclusions occurred before laboratory analysis, and

• whether the excluded participants differed meaningfully from those included.

A supplementary table comparing excluded and retained participants would substantially strengthen transparency.

5. Address referral-source imbalance as a potential source of bias

Cases were substantially more likely to have been referred from peripheral facilities than controls (78% versus 39%). This difference could reflect variation in antenatal care, supplementation practices, disease severity, or other factors independent of vitamin D status.

This issue deserves explicit discussion as a potential source of selection bias. If feasible, a sensitivity analysis adjusting for or stratifying by referral status would strengthen confidence in the reported association.

6. Expand discussion of reverse causation

A major alternative explanation currently receives insufficient attention.

Because proteinuria is part of the diagnostic criteria for pre-eclampsia, urinary loss of vitamin D-binding protein may reduce circulating total 25(OH)D concentrations. Consequently, lower vitamin D levels may represent a consequence rather than a cause of established disease.

Although this cannot be fully addressed within the current study, this mechanism should be discussed more explicitly as an important limitation. If vitamin D-binding protein or free/bioavailable vitamin D measurements are available, their inclusion would considerably strengthen the manuscript.

7. Clarify generalizability

Only pregnancies at or beyond 28 weeks were eligible for inclusion. Consequently, early-onset pre-eclampsia—which has distinct pathophysiological mechanisms—was excluded.

Please acknowledge that the findings primarily apply to late-onset disease and that extrapolation to all forms of pre-eclampsia should be made cautiously.

8. Expand discussion of unmeasured confounding

The absence of information regarding maternal vitamin D supplementation, multivitamin use, skin pigmentation, and detailed sun exposure represents an important limitation. Supplementation, in particular, could substantially influence serum vitamin D concentrations independent of disease status and should receive greater emphasis in the Discussion.

9. Consider additional analyses

If feasible, the manuscript would be strengthened by:

• sensitivity analyses excluding referred participants,

• multivariable adjustment including BMI, anemia, and referral status,

• clarification of missing-data handling within matched analyses,

• exploration of potential non-linearity in the continuous vitamin D model (for example, restricted cubic splines), and

• consideration of exact conditional logistic regression for sparse vitamin D deficiency categories.

While some analyses may not be feasible because of sample size, discussing these limitations would improve interpretation.

10. Update the literature review

The literature review would benefit from incorporation of more recent evidence.

In particular, please consider citing the 2024 systematic review and meta-analysis by Moghib et al., which included 33 randomized controlled trials and reported that vitamin D supplementation significantly reduced the incidence of pre-eclampsia. This provides an important update to the evidence currently cited and would strengthen both the Introduction and Discussion.

You may also wish to consider relevant Cochrane evidence and current WHO recommendations regarding vitamin D supplementation during pregnancy to better contextualize your conclusions.

Interpretation and Discussion

Overall, the Discussion is balanced and appropriately acknowledges many limitations. Nevertheless, several statements should be interpreted more cautiously.

• Because of the observational case-control design, terms such as "risk factor" should be replaced with "risk marker" or "associated factor" where appropriate.

• The statement describing the OR at the <75 nmol/L threshold as "borderline significant" should be revised to indicate that the association was not statistically significant, while noting that the direction of effect was consistent with the primary findings.

• The Discussion should explicitly acknowledge reverse causation, referral-source imbalance, exclusion of early-onset disease, and unmeasured supplementation as important limitations.

Minor Comments

1. Standardize capitalization of pre-eclampsia throughout according to journal style.

2. Revise the sentence "This was inflated to 120 participants" for clarity.

3. Correct the phrase "Differences in were compared...".

4. Standardize the abbreviation NACOSTI throughout the manuscript.

5. Correct the typo MRTH to MTRH.

6. Correct the spacing error in Table 1 ("11. 9").

7. Add footnotes indicating which statistical tests were used for BMI and anemia.

8. Ensure all vitamin D concentrations consistently include units (nmol/L).

9. Verify consistency of reference numbering after inclusion of additional citations.

10. Clarify Figure 2 axis labels and improve consistency between panels.

11. Consider reporting exact p-values rather than "<0.001" where appropriate.

12. Clarify Table 3 footnotes regarding adjusted versus unadjusted odds ratios.

13. Ensure all supplementary files are correctly named and referenced.

14. Confirm that the STROBE checklist is complete and appropriately cross-referenced.

Overall Recommendation

This manuscript addresses an important research question and contributes valuable evidence from a region where data remain limited. The study has several methodological strengths, including prospective registration, blinded laboratory assessment, transparent data sharing, and a matched case-control design. However, several important statistical, methodological, and reporting issues should be addressed before the findings can be interpreted with confidence. In particular, the numerical inconsistencies, clarification of matched statistical analyses, discussion of reverse causation, transparency regarding participant exclusions, 2wsand more explicit consideration of potential sources of bias require revision.

Overall, I believe these concerns are largely addressable, and I encourage the authors to revise the manuscript accordingly. Following satisfactory revision, this work has the potential to make a meaningful contribution to the literature on maternal vitamin D status and pre-eclampsia in sub-Saharan Africa.

**********

what does this mean?). If published, this will include your full peer review and any attached files.

If you choose “no”, your identity will remain anonymous but your review may still be made public.

**Do you want your identity to be public for this peer review?**  For information about this choice, including consent withdrawal, please see our Privacy Policy

Reviewer #1: **Yes:** G. Iatrakis

Reviewer #2: **Yes:** IZERE Salomon

**********

[NOTE: If reviewer comments were submitted as an attachment file, they will be attached to this email and accessible via the submission site. Please log into your account, locate the manuscript record, and check for the action link "View Attachments". If this link does not appear, there are no attachment files.]

To ensure your figures meet our technical requirements, please review our figure guidelines: https://journals.plos.org/plosone/s/figures

You may also use PLOS’s free figure tool, NAAS, to help you prepare publication quality figures: https://journals.plos.org/plosone/s/figures#loc-tools-for-figure-preparation.

NAAS will assess whether your figures meet our technical requirements by comparing each figure against our figure specifications.

# Author comment: Author response to Decision Letter 1

**DOI:** 10.1371/journal.pone.0358781.r002

10 Aug 2026

Response to Reviewers

Manuscript number: PONE-D-26-14449

Manuscript title: Maternal Serum Vitamin D Levels and Preeclampsia among Kenyan Pregnant Women: a matched case-control study

Corresponding author: Kimbley Asaso Omwodo

________________________________________

Dear Dr Magnus, Reviewer 1, and Reviewer 2,

Thank you for the careful and constructive evaluation of our manuscript. We have revised the manuscript.

Journal requirements

1. PLOS ONE style and file naming

Please ensure that your manuscript meets PLOS ONE’s style requirements, including those for file naming.

Response: We have revised the manuscript and file names to conform to PLOS ONE style. The revision package uses separate files for the unmarked manuscript, the tracked-changes manuscript, this response letter, Figure 1, Figure 2, and consecutively named Supporting Information files. In-text Supporting Information citations and end-of-manuscript captions have been aligned with the file names.

2. Abstract consistency

Please amend either the abstract on the online submission form or the abstract in the manuscript so that they are identical.

Response: The abstract in the online submission form has been replaced with the revised manuscript abstract verbatim. The corrected numerical values and cautious interpretation are identical in both locations.

3. Potentially identifying data in Supplementary 3

Please remove or anonymize all personal information, ensure that the data shared are in accordance with participant consent, and re-upload a fully anonymized data set. Spreadsheet columns with personal information must be removed and not hidden.

Response: The original spreadsheet has been withdrawn. The replacement dataset has been reviewed against the ethics approval and participant information/consent documents. Only variables required to reproduce the analyses have been retained.

4. Supporting Information captions

Please include captions for your Supporting Information files at the end of your manuscript, and update any in-text citations to match accordingly.

Response: A Supporting Information section has been added at the end of the manuscript. In-text references now use the corresponding S-file designations.

Supporting information

S1 File. STROBE checklist.

S2 File. Additional statistical analyses. Collinearity diagnostics and multivariable conditional logistic regression models evaluating adjustment for body mass index, referral status, and anemia.

S3 File. Fully anonymized analytic dataset.

Additional Editor Comments

Several issues have been highlighted. If your manuscript is to be further considered for publication, you need to carefully address all comments provided by the reviewers. In particular, you need to carefully assess the manuscript for any inconsistencies as pointed out.

Response: We conducted a full consistency check across the Abstract, Results, Tables 1–3, figure captions, STROBE checklist, and Supplementary 2.

Reviewer 1

This is an interesting study. However, considering that the findings and the conclusions of this study are almost similar to those of Reference 23, some more comments are necessary in your manuscript about the “originality” of your findings.

Response: We appreciate this important observation and have substantially revised the manuscript to clarify the distinct intellectual and empirical contributions of our study relative to Reference 23. We agree that without explicit framing, the relationship between a primary observational cohort and a secondary evidence synthesis could appear overlapping. Our aim was to address the regional evidence gaps identified in prior reviews and providing context-specific estimates.

We had noted (line 83 -89): ‘’A systematic review in 2020 that included pooled data from twenty-seven randomised controlled trials (RCTs) involving 4,777 participants reported that vitamin D supplementation resulted in a 63% risk reduction in the occurrence of PE (8). Even so, the studies included in the review did not strictly select participants on the basis of vitamin D status, nor did they evaluate the achievement of optimum vitamin D levels post-supplementation. The authors also noted that the results could not be generalised to an African population, given the absence of studies that included this cohort.’’

Reference 23 addresses the question: Does vitamin D supplementation reduce preeclampsia incidence in pooled trial populations? Our study addresses a different, complementary question: What is the observed association between antenatal serum 25(OH)D levels and PE risk in a Kenyan cohort? A critical limitation of Reference 2 (and a major justification for our study) is that none of the 27 pooled RCTs included participants from sub-Saharan Africa.

Revision made: We have added the following text to the Discussion:

Line 377: ‘’The outcomes also add to the data necessary to evaluate whether vitamin D assessment warrants integration into pre-eclampsia risk stratification protocols in Kenyan antenatal care. This represents an incremental contribution to literature.’’

We also emphasize that the study Conclusion is presented as identifying an association and generating hypotheses, not as independently establishing a preventive effect.

Reviewer 2

We address each point below.

Major comments

1. Internal numerical inconsistencies require correction

Several discrepancies were identified between the Abstract, Results section, and Table 2. These should be reconciled against the original dataset before publication.

• The reported interquartile ranges (IQRs) for maternal serum vitamin D differ across the Abstract, Results narrative, and Table 2.

• The number and percentage of controls classified as vitamin D sufficient are inconsistent. The Results text reports 54 (91.5%), whereas Table 2 reports 55 (93.2%). Internal arithmetic supports the Table 2 values.

• The reported prevalence of suboptimal vitamin D among controls is inconsistently presented as 6.8% and 6.9%. Please standardize rounding throughout the manuscript.

Because these discrepancies affect confidence in the reported findings, all numerical values should be carefully verified and made fully consistent across the manuscript.

Response: We thank the reviewer for this meticulous scrutiny. We have re-calculated all numerical values directly from the final locked dataset and identified the source of each discrepancy.

All inconsistencies arose from transcription errors during manuscript drafting (copying values between software output to the Abstract, and Table 2). None of the errors alter the study conclusions. We have made the necessary revisions.

2. Clarify the statistical analyses used for matched data

The manuscript describes a matched case-control study; however, several statistical procedures appear inconsistent with the matched design.

• The Methods and Table 2 indicate use of the Mann–Whitney U (Wilcoxon rank-sum) test for skewed continuous variables. Since observations are matched, the appropriate non-parametric paired alternative would generally be the Wilcoxon signed-rank test. If the rank-sum test was intentionally used, please justify this approach or consider reanalysis using methods appropriate for matched data.

• Similarly, Table 1 reports baseline categorical comparisons using conditional logistic regression likelihood-ratio tests. This approach is unusual for descriptive baseline comparisons in matched studies. Please clarify the rationale or consider using conventional matched categorical tests where appropriate.

Response: We sincerely thank the reviewer for this expert methodological critique. We have re-analyzed all comparisons using tests appropriate for 1:1 matched data, and we have revised the Methods, Table 1, and Table 2 accordingly. Below, we address each concern in turn.

1. Mann–Whitney U test for vitamin D comparison in Table 2: We acknowledge this was a methodological error. We re-ran the comparison of serum 25(OH)D concentrations between the 59 matched case-control pairs using the Wilcoxon signed-rank test in Stata 19.0 (signrank command). The corrected result is: Wilcoxon signed-rank test: Z = 3.45, P = 0.001 (two-sided)

Revisions made:

Methods (page 5, line 207): “Wilcoxon signed-rank tests for skewed continuous variables”

Table 2 footnote: Changed “P-value derived from Wilcoxon signed-rank test for matched pairs.”

We have removed the p-values for parity from Table 1. Parity was a matching factor (matched as 0, 1, or ≥2); we agree that testing for “balance” in a matched variable is statistically circular and adds no valid information. We have added a footnote: “Parity was matched by design (0, 1, or ≥2); no formal test of balance is reported.”

For age and gestational age, the paired t-tests are retained. McNemar’s exact test is maintained for non-matched binary variables.

We have replaced the conditional logistic regression p-values with Stuart–Maxwell tests for marginal homogeneity (implemented in Stata via the symmetry package). The corrected p-values have been updated in table 1. All corrected p-values remain non-significant (all >0.05), confirming that the groups were well-balanced on these sociodemographic characteristics.

Revisions made:

Table 1: Removed p-value for parity; replaced p-values for education, work status, marital status, and ANC visits with Stuart–Maxwell test results.

Table 1 footnotes: Updated to read: “*P-value from Stuart–Maxwell test for marginal homogeneity in matched pairs (polytomous categorical variables).” and “‡ P-value from paired t-test (continuous matched variables).”

3. Remove the post hoc power calculation

The reported post hoc (achieved) power calculated from the observed effect size does not provide meaningful additional information and is generally discouraged because it is mathematically related to the observed p-value. Instead, I recommend removing this analysis and emphasizing confidence intervals and the precision of the estimates.

Response: We have removed the post hoc achieved-power statement. The Study Size section now retains only the a priori pilot-study rationale and target sample size.

4. Clarify exclusion of six enrolled participants

The manuscript states that six participants were excluded after a post hoc audit of the protocol-specified matching criteria. Additional transparency is needed.

Please clarify:

• precisely how "unmatched" was defined,

• why matching was not resolved during recruitment,

• whether exclusions occurred before laboratory analysis, and

• whether the excluded participants differed meaningfully from those included.

A supplementary table comparing excluded and retained participants would substantially strengthen transparency.

Response: We thank the reviewer for prompting deeper transparency.

"Unmatched" was defined as any enrolled participant who could not be paired 1:1 with an opposite group participant (case with control, or control with case) according to the three protocol-specified matching criteria. That is maternal age (±3 years), gestational age at delivery (±1 week) and Parity category (0, 1, or ≥2). All three criteria had to be satisfied simultaneously for a valid pair. The matching audit was conducted post-hoc after enrollment closed, using the final cleaned dataset.

Of 124 enrolled participants (62 cases, 62 controls), 6 were unmatched:

� 3 cases had no eligible control in the concurrently recruited pool.

� 3 controls could not be matched to any remaining case.

The final analytic sample therefore comprised 59 matched case–control pairs (n = 118). Matching could not be resolved during recruitment because eligible counterparts were either absent from the ward at the time of enrollment or had already been enrolled and matched to another participant.

Exclusions occurred after laboratory analysis. All 6 unmatched participants have valid serum 25(OH)D concentrations and laboratory tracking IDs in the dataset. The post-hoc matching audit was conducted using demographic and clinical data only; the audit was blinded to vitamin D results to prevent selective exclusion based on exposure status.

We compared the 6 excluded participants to the 118 included participants on all baseline characteristics. The results are presented in the new Supplementary File 2.

The excluded participants were broadly similar to those included, with two notable differences:

� Excluded participants had a slightly lower mean gestational age (31.7 vs. 35.4 weeks).

� All 6 excluded participants were parity 0 or 1; no parity ≥2 participants were excluded.

Excluded cases did not have systematically lower (or higher) vitamin D levels than included cases. The three excluded cases had vitamin D concentrations spanning the full range (36.2, 48.7, and 64.4 nmol/L), and the excluded controls included both the highest (129.8 nmol/L) and one of the lowest (39.9 nmol/L) values in the entire cohort. This confirms that exclusion was not differential by vitamin D status.

We have added in Results section of manuscript (line 249): "Six enrolled participants (3 cases, 3 controls) were excluded post-hoc because no eligible 1:1 match could be identified. Excluded participants did not differ systematically from included participants by demographic characteristics or vitamin D status (Supplementary File 2)."

5. Address referral-source imbalance as a potential source of bias

Cases were substantially more likely to have been referred from peripheral facilities than controls (78% versus 39%). This difference could reflect variation in antenatal care, supplementation practices, disease severity, or other factors independent of vitamin D status.

This issue deserves explicit discussion as a potential source of selection bias. If feasible, a sensitivity analysis adjusting for or stratifying by referral status would strengthen confidence in the reported association.

Response: We thank the reviewer for this important observation. We agree that the referral imbalance warrants explicit discussion and transparency. However, we respectfully point out that referral status is a post-diagnosis consequence of disease severity, adjusting for it in the primary model would introduce over-adjustment bias.

The causal timeline in our study is: Vitamin D status (pre-existing) → Pre-eclampsia develops → Severe PE triggers referral to MTRH → Enrollment.

Moi Teaching and Referral Hospital is a tertiary referral centre; women are referred from peripheral facilities because they have developed severe or complicated pre-eclampsia requiring specialist management. Referral occurs after the outcome has manifested. It is therefore a post-diagnosis variable (or mediator), not a pre-exposure confounder. The referral-adjusted model is however included in supplementary file 2 as aOR 8.34.

To address the reviewer's request for transparency without committing over-adjustment bias, we performed a stratified analysis by referral concordance within matched pairs.

We have also added this in the Supplementary file 2 and referenced it in the Results (line 322):

"A stratified analysis by referral concordance within matched pairs showed that the maternal serum 25(OH)D concentration and PE association was present in both referred and non-referred subgroups (Supplementary File 2).”

6. Expand discussion of reverse causation

A major alternative explanation currently receives insufficient attention.

Because proteinuria is part of the diagnostic criteria for pre-eclampsia, urinary loss of vitamin D-binding protein may reduce circulating total 25(OH)D concentrations. Consequently, lower vitamin D levels may represent a consequence rather than a cause of established disease.

Although this cannot be fully addressed within the current study, this mechanism should be discussed more explicitly as an important limitation. If vitamin D-binding protein or free/bioavailable vitamin D measurements are available, their inclusion would considerably strengthen the manuscript.

Response: Our manuscript states cautiously that suboptimal vitamin D status is associated with preeclampsia, rather than implying a causal relationship. We have however expanded the Discussion to address reverse causation and the urinary loss of VDBP in PE.

Several lines of evidence from the broader literature support the interpretation that vitamin D deficiency can antedate preeclampsia onset. Bodnar et al. conducted a nested case-control study within a prospective cohort and found that maternal 25(OH)D insufficiency in early pregnancy (prior to 22 weeks) was associated with a 5-fold increased risk of preeclampsia, with a dose-response relationship. Baker et al. similarly demonstrated that mid-gestation 25(OH)D deficiency preceded the development of severe preeclampsia, noting that "maternal serum was collected well before delivery at a time when no clinical manifestations of preeclampsia were evident, reducing the likelihood that subclinical disease affected vitamin D levels."

In the VDAART randomized trial, sufficient vitamin D status (25(OH)D ≥30 ng/mL) at trial entry (10–18 weeks) was associated with lower preeclampsia risk compared with insufficient levels, and differential expression of vitamin D-associated immune transcripts was already detectable in early pregnancy among women who later developed preeclampsia

Revision made:

Discussion and Strengths and Limitations: We have expanded the existing limitation statement to explicitly acknowledge the VDBP mechanism:

Line 345: ‘’An alternative explanation for the observed association is reverse causation. Because proteinuria is integral to the diagnosis of PE, glomerular injury may increase urinary loss of vitamin D-binding protein (VDBP) (26). Reduced measured total 25(OH)D concentrations could be a consequence of established disease rather than as a predisposing factor.

Nevertheless, prospective studies that measured 25(OH)D in early or mid-gestation have consistently reported that lower vitamin D status precedes disease onset, supporting a temporal sequence in which deficiency may contribute to pathogenesis (20, 27).’’

Line 367: ‘’Determination of whether deficiency preceded PE onset could not be determined. The observed association should therefore be viewed as hypothesis-generating rather than evidence of causality.’’

7. Clarify generalizability

Only pregnancies at or beyond 28 weeks were eligible for inclusion. Consequently, early-onset pre-eclampsia—which has distinct pathophysiological mechanisms—was excluded.

Please acknowledge that the findings primarily apply to late-onset disease and that extrapolation to all forms of pre-eclampsia should be made cautiously.

Response We thank the reviewer for this observation. The manuscript already notes that vitamin D was measured at a single time point at or beyond 28 weeks and that the single-center design may limit generalizability (Discussion, Strengths and limitations). Early-onset preeclampsia is clinically defined as onset before 34 weeks' gestation (by major guidelines including ISSHP, ACOG, and NICE). Our eligibility criterion (≥28 weeks) permitted enrollment of women diagnosed at 28–33 weeks. In our dataset, 12 cases (19.4%) were enrolled at <32 weeks and 24 cases (38.7%) at 32–36 weeks, meaning that at minimum nearly one-fifth of cases represent definitively early-onset disease.

We have made the following addition in the Strengths and limitations paragraph, line 368:

" Our findings also only primarily apply to PE presenting at or beyond 28 weeks."

8. Expand discussion of unmeasured confounding

The absence of information regarding maternal vitamin D supplementation, multivitamin use, skin pigmentation, and detailed sun exposure represents an important limitation. Supplementation, in particular, could substantially influence serum vitamin D concentrations independent of disease status and should receive greater emphasis in the Discussion.

Response: We thank the reviewer for this important observation. The original manuscript stated that we were "unable to assess potential confounders including seasonality and skin pigmentation intensity." We have revised this to accurately reflect which variables were collected and why they were not retained in the primary analysis. The following addition has been made to the Strengths and limitations paragraph:

Line 371: ‘’Self-reported data on vitamin D supplementation, prenatal multivitamin use, daily outdoor hours, and sunscreen use were collected. These variables were however not included in the primary conditional logistic regression model. Vitamin D supplementation was infrequent with 4 of 124 participants reporting any use. All were at less than 400 IU per day. Prenatal multivitamin use was also uncommon, with minimal case-control imbalance. They also exhibited high collinearity with matching factors. Skin pigmentation intensity was not quantified. It remains a genuine unmeasured confounder. Seasonality was mitigated by recruiting across both dry and wet seasons. It was not included as an analytical covariate.’’

9. Consider additional analyses

If feasible, the manuscript would be strengthened by:

• sensitivity analyses excluding referred participants,

• multivariable adjustment including BMI, anemia, and referral status,

• clarification of missing-data handling within matched analyses,

• exploration of potential non-linearity in the continuous vitamin D model (for example, restricted cubic splines), and

• consideration of exact conditional logistic regression for sparse vitamin D deficiency categories.

While some analyses may not be feasible because of sample size, discussing these limitations would improve interpretation.

Response: We thank the reviewer for these constructive suggestions. Several of the requested analyses have already been performed and are reported in Supplementary File 2 (Additional Analysis). Others are constrained by sample size. We address each point below.

1. Multivariable adjustment including BMI, anemia, and referral status: This has been completed and is presented in Supplementary File 2, Table 5. We fitted three conditional logistic regression models:

Model 1: Suboptimal vitamin D (<50 nmol/L) + BMI (n=116)

Model 2: Suboptimal vitamin D + BMI + referred status (n=116)

Model 3: Suboptimal vitamin D + BMI + referred status + anemia (n=116)

The adjusted odds ratios were: Model 1 aOR 14.30 (95% CI 1.87–109.57); Model 2 aOR 8.34 (1.02–68.44); Model 3 aOR 13.61 (1.37–135.00). Collinearity diagnostics confirmed all VIFs <1.1 (Supplementary File 2, Table 4).

2. Sensitivity analysis excluding referred participants: Only 12 complete matched pairs (24 participants) had both members recruited directly at MTRH without referral. Excluding all referred participants would reduce the analytic sample by 79% and leave only 15 non-referred cases versus 38 non-referred controls. With 12 complete pairs, statistical power would fall below 50%. We therefore retained referral status as a covariate in multivariable adjustment (Model 2) rather than performing a complete-case exclusion sensitivity analysis.

3. Clarification of missing-data handling: Missing data were handled by complete case analysis. In the matched design, pairs with missing covariate data in either member were excluded from adjusted models. Four participants (2 pairs) had missing BMI data. These pairs were excluded from Models 1–3, reducing the adjusted sample from 118 to 116. We have added this clarification to the

Methods Line 212: "Missing data were handled by complete case analysis; matched pairs with missing covariate data in either member were excluded from adjusted models."

4. Exploration of non-linearity (restricted cubic splines): This is not feasible with the current sample. Restricted cubic splines require adequate event counts across the exposure distribution to estimate knot-specific slopes reliably. With only 59 matched pairs and 6 participants (5 cases, 1 control) in the <30 nmol/L category, spline models would be unstable and prone to overfitting. We have added this as a stated limitation.

5. Exact conditional logistic regression for sparse categories: The <30 nmol/L category contains only 6 participants total (5 cases, 1 control). We fitted exact conditional logistic regression for this category. The exact OR was 5.0 (95% exact CI: 0.28–89.1; p=0.29). As expected given the extreme sparsity, precision did not improve materially beyond the standard asymptotic estimate (OR 5.0, 95% CI 0.58–42.8).

Revised text for the manuscript

We have revised the Results (Line 313) to add:

" Multivariable adjustment for BMI, and anemia did not materially alter the association between suboptimal vitamin D and PE (Supplementary File 2). However, the small sample size precluded formal exploration of non-linear dose-response relationships via restricted cubic splines. Exact conditional logistic regression for the sparse <30 nmol/L category yielded an OR of 5.0 (exact 95% CI 0.28–89.1) (Supplementary File 2).’’

We believe these revisions and the existing supplementary analyses adequately address the reviewer's requests.

10. Update the literature review

The literature review would benefit from incorporation of more recent evidence.

In particular, please consider citing the 2024 systematic review and meta-analysis by Moghib et al., which included 33 randomized controlled trials and reported that vitamin D supplementation significantly reduced the incidence of pre-eclampsia. This provides an important update to the evidence currently cited and would strengthen both the Introduction and Discussion.

You may also wish to consider relevant Cochrane evidence and current WHO recommendations regarding vitamin D supplementation during pregnancy to better contextualize your conclusions.

Interpretation and Discussion

Overall, the Discussion is balanced and appropriately acknowledges many limitations. Nevertheless, several statements should be interpreted more cautiously.

• Because of the observational case-control design, terms such as "risk factor" should be replaced with "risk marker" or "associated factor" where appropriate.

• The statement describing the OR at the <75 nmol/L threshold as "borderline significant" should be revised to indicate that the association was not statistically significant, while noting that the direction of effect was consistent with the primary findings.

• The Discussion should explicitly acknowledge reverse causation, referral-source imbalance, exclusion of early-onset disease, and unmeasured supplementation as important limitations.

Response:

We thank the reviewer for these important suggestions. We have made the following revisions across the manuscript.

A. Literature update

1. Moghib et al. 2024 citation has been added to both the Introduction and Discussion. It provides an update to the Fogacci et al. 2020 meta-analysis (27 RCTs, n=4,777, 63% risk reduction) currently cited in the Introduction.

2. We have added the 2024 Cochrane review by Palacios et al. to contextualize the uncertainty in the trial evidence. Following trustworthiness screening that removed 21 studies from the previous update, the 2024 review included only 10 studies and found very uncertain evidence on the effect of vitamin D alone versus placebo or no intervention on preeclampsia (RR 0.53, 95% CI 0.21–1.33; 1 study, 165 women; very low-certainty evidence). The review concluded that most findings warrant further research and that additional rigorous, high-quality trials are required.

3. We have added the 2023 WHO guidance, which states: "Oral vitamin D supplementation is not recommended for all pregnant women to improve maternal and perinatal outcomes." This recommendation updates but does not alter the respective 2016 WHO antenatal care guideline. We have revised the Discussion to note that our findings should be interpreted in light of this guidance.

Revised Introduction paragraph:

Line 90: ‘’A more recent meta-analysis of 33 RCTs (n=10,613) similarly reported a 45% reduction in preeclampsia risk with supplementation (9). Notably, the analysis included only one study from Africa (Congo).

Revised Discussion paragraph:

Line 366: ‘’A 2024 meta-analysis of 33 RCTs similarly reported a 45% reduction (9). However, a 2024 Cochrane review of 10 studies involving 2313 pregnant women found only very low-certainty evidence for vitamin D supplementation on PE, gestational diabetes, preterm birth, or nephritic syndrome (30). Currently, the World Health Organization (WHO) does not recommend universal vitamin D supplementation in pregnancy (31).’’

B. Cautious interpretation of findings

1. Replacement of "risk factor" with "associated factor" / "risk marker"

Line 135 (Methods): "The selected matching factors were established risk factors for PE" → "The selected matching factors were established risk factors for PE" [Retained here because maternal age, parity, and gestational age are indeed established risk factors for PE from prospective cohort evidence, independent of this study's design.]

Line 328 (Discussion): "suboptimal levels as a particularly important risk factor" → "suboptimal levels as a particularly important associated factor"

Line 328 (Discussion): "among women with risk of PE" → "among women at risk of PE" [Retained because "at risk" is descriptive, not causal.]

Discussion, line 331: "A significant association was seen between low maternal vitamin D levels and odds of PE" [Already appropriately cautious.]

2. Revision of "borderline significance"

The statement at line 303: "At the <75 nmol/L threshold, the association reduced to borderline significance (OR 2.0; 95% CI: 0.89–4.78; p=0.068)" has been revised to:

"At the <75 nmol/L threshold, the association was not statistically significant (OR 2.0; 95% CI: 0.89–4.78; p=0.068), though the direction of effect was consistent with the primary findings."

C. Explicit acknowledgment of limitations

These have been addressed in the revised Strengths and limitations paragraph.

Minor Comments

1. Standardize capitalization of pre-eclampsia throughout according to journal style.

2. Revise the sentence "This was inflated to 120 participants" for clarity.

3. Correct the phrase "Differences in were compared...".

4. Standardize the abbreviation NACOSTI throughout the manuscript.

5. Correct the typo MRTH to MTRH.

6. Correct the spacing error in Table 1 ("11. 9").

7. Add footnotes indicating which statistical tests were used for BMI and anemia.

8. Ensure all vitamin D concentrations consistently include units (nmol/L).

9. Verify consistency of reference numbering after inclusion of additional citations.

10. Clarify Figure 2 axis labels and improve consistency between panels.

11. Consider reporting exact p-values rather than "<0.001" where appropriate.

12. Clarify Table 3 footnotes regarding adjusted versus unadjusted odds ratios.

13. Ensure all supplementary files are correctly named and referenced.

14. Confirm that the STROBE checklist is complete and appropriately cross-referenced.

Response: We thank the reviewer for these detailed editorial observations. We have addressed each point systematically.

1. Standard adopted: "preeclampsia" (no hyphen, lowercase in text). All instances corrected.

2. Corrected: "The target sample size was inflated to 120 participants to account for potential attrition."

3. Corrected: "Differences in continuous variables were compared using paired t-tests for normally distributed data, Wilcoxon signed-rank tests for skewed data, and McNemar's test or Fisher's exact test for categorical variables."

4. Corrected: "National Commission for Science, Technology, and Innovation (NACOSTI) Kenya"

5. Corrected: "Written authorisation was obtained from the management of MTRH"

6. Spacing error "11. 9" in Table 1. Corrected.

7. BMI p-value cell updated to read "0.319‡" to match the footnote.

8. All concentration values already include units. Clarification added on:

Abstract (line 26): "Serum 25-hydroxyvitamin D [25(OH)D] levels" → "Serum 25-hydroxyvitamin D [25(OH)D] concentrations (nmol/L)" [on first mention in Results section]

All values in Results, Tables 2–3, and Figure 2 legend consistently include nmol/L.

9. Three new references added. Reference list updated

10. Corrected caption: "Panel A: Slope graph of individual serum 25(OH)D concentrations for each matched pair. Red lines indicate preeclampsia cases and grey lines indicate controls. Panel B: Paired values with grey connecting lines. Green dashed lines represent the 50 nmol/L threshold. Orange dashed lines represent the 30 nmol/L threshold."

11. All p-values in Table 3 and continuous model are already reported as exact values. Reported p-value <0.001 is because software output is truncated at this threshold.

12. Corrected footnote: "*The <30 nmol/L estimate was derived from exact conditional logistic regression due to sparse data (6 events); all other estimates are from standard conditional logistic regression accounting for matched pairs (maternal age ±3 years, gestational age ±1 week, parity). No additional covariate adjustment was applied."

We believe this clarifies that (a) all ORs account for matching, (b) the <30 nmol/L row used exact methods due to sparsity, and (c) Table 3 presents crude matched analyses and multivariable adjusted models are in Supplementary File 2.

13. Manuscript references standardized:

File Manuscript reference File name

STROBE checklist Supplementary File 1 " S1_File.pdf — STROBE Checklist"

Additional analyses Supplementary File 2 " S2_File.pdf — Additional Analysis"

De-identified dataset Supplementary File 3 " S3_File.xlsx — Fully Anonymized Dataset"

14. The completed STROBE checklist is provided as Supplementary File 1.

Attachment Submitted filename: Response to Reviewers.docx

# Peer review: Decision Letter 1

**DOI:** 10.1371/journal.pone.0358781.r003

**Authors:** Maria Magnus

26 Aug 2026

Dear Dr. Asaso Omwodo,

Thank you for submitting your manuscript to PLOS One. After careful consideration, we feel that it has merit but does not fully meet PLOS One’s publication criteria as it currently stands. Therefore, we invite you to submit a revised version of the manuscript that addresses the points raised during the review process.

Please submit your revised manuscript by Oct 10 2026 11:59PM. If you will need more time than this to complete your revisions, please reply to this message or contact the journal office at plosone@plos.org. When you're ready to submit your revision, log on to https://www.editorialmanager.com/pone/ and select the 'Submissions Needing Revision' folder to locate your manuscript file.

- A letter that responds to each point raised by the academic editor and reviewer(s). You should upload this letter as a separate file labeled 'Response to Reviewers'.
- A marked-up copy of your manuscript that highlights changes made to the original version. You should upload this as a separate file labeled 'Revised Manuscript with Track Changes'.
- An unmarked version of your revised paper without tracked changes. You should upload this as a separate file labeled 'Manuscript'.

If you would like to make changes to your financial disclosure, please include your updated statement in your cover letter. Guidelines for resubmitting your figure files are available below the reviewer comments at the end of this letter.

If applicable, we recommend that you deposit your laboratory protocols in protocols.io to enhance the reproducibility of your results. Protocols.io assigns your protocol its own identifier (DOI) so that it can be cited independently in the future. For instructions see: https://journals.plos.org/plosone/s/submission-guidelines#loc-laboratory-protocols. Additionally, PLOS One offers an option for publishing peer-reviewed Lab Protocol articles, which describe protocols hosted on protocols.io. Read more information on sharing protocols at https://plos.org/protocols?utm_medium=editorial-email&utm_source=authorletters&utm_campaign=protocols.

As the corresponding author, your ORCID iD is verified in the submission system and will appear in the published article. PLOS supports the use of ORCID, and we encourage all coauthors to register for an ORCID iD and use it as well. Please encourage your coauthors to verify their ORCID iD within the submission system before final acceptance, as unverified ORCID iDs will not appear in the published article. *Only*  the individual author can complete the verification step; PLOS staff *cannot*  verify ORCID iDs on behalf of authors.

We look forward to receiving your revised manuscript.

Kind regards,

Maria Christine Magnus, PhD

Academic Editor

PLOS One

Journal Requirements:

If the reviewer comments include a recommendation to cite specific previously published works, please review and evaluate these publications to determine whether they are relevant and should be cited. There is no requirement to cite these works unless the editor has indicated otherwise.

Please review your reference list to ensure that it is complete and correct. If you have cited papers that have been retracted, please include the rationale for doing so in the manuscript text, or remove these references and replace them with relevant current references. Any changes to the reference list should be mentioned in the rebuttal letter that accompanies your revised manuscript. If you need to cite a retracted article, indicate the article’s retracted status in the References list and also include a citation and full reference for the retraction notice.

Additional Editor Comments :

The revised version of your manuscript have now been reviewed again by the initial peer reviewers. One of the reviewers has highlihgted some remaining concerns. I therefore ask that you carefully adress these remaining comments. Pay particular attention to the frequencies and ensure that all numbers presented are correct and add up to expected values.

[Note: HTML markup is below. Please do not edit.]

Reviewers' comments:

Reviewer's Responses to Questions

**Comments to the Author**

Reviewer #1: All comments have been addressed

Reviewer #2: (No Response)

**********

2. Is the manuscript technically sound, and do the data support the conclusions?

Reviewer #1: Yes

Reviewer #2: Partly

**********

3. Has the statistical analysis been performed appropriately and rigorously?

Reviewer #1: Yes

Reviewer #2: Yes

**********

4. Have the authors made all data underlying the findings in their manuscript fully available?

The PLOS Data policy

Reviewer #1: Yes

Reviewer #2: Yes

**********

5. Is the manuscript presented in an intelligible fashion and written in standard English?

Reviewer #1: Yes

Reviewer #2: Yes

**********

Reviewer #1: Reviewer's comments were taken into account and the paper can now be published. Reviewer's comments were taken into account and the paper can now be published.

Reviewer #2: I appreciate the considerable effort invested in revising this manuscript. The revised version demonstrates a meaningful improvement in methodological rigor, statistical appropriateness, and transparency. The study addresses an important clinical question in a population where evidence remains limited.

Before the manuscript can be accepted, I recommend addressing the following remaining issues.

Major Comments

1. Correct the remaining arithmetic inconsistency in Table 2 (Mandatory)

The manuscript still contains one unresolved numerical inconsistency in Table 2.

The control group vitamin D categories currently report:

Deficient = 1

Insufficient = 3

Sufficient = 54

These values sum to 58, whereas the control sample size is reported as 59. Consequently, the overall total also sums to 117 instead of 118.

Based on the reported suboptimal vitamin D counts elsewhere in the manuscript, the internally consistent values should be:

- Controls: 55 participants (93.2%) with sufficient vitamin D.

- Overall: 95 participants (80.5%) with sufficient vitamin D.

Please correct Table 2 and the corresponding sentence in the Results section and verify that all percentages are recalculated using the correct denominators.

2. Verify the originality argument after reference renumbering (Mandatory)

Minor Comments

3. Update the Ethics Statement metadata

The manuscript text appears to have been corrected, but the submission metadata reportedly still contains the previous terminology ("MRTH" and "NASCOTI").

Please ensure that the Ethics Statement in the submission system matches the manuscript text by using MTRH and NACOSTI consistently.

4. Reconcile the Supporting Information file format

5. Revise the statement regarding supplementation and collinearity

The manuscript states that vitamin D supplementation or multivitamin use exhibited "high collinearity with matching factors." This assertion is not supported by any statistical evidence presented in the manuscript.

Unless supporting diagnostics are provided, I recommend replacing this statement with a simpler explanation indicating that supplementation was too infrequent to be modeled reliably. Also clarify that the supplementation denominator refers to the 124 enrolled participants, whereas the analytical sample consisted of 118 matched participants.

6. Ensure consistency in the multivariable adjustment description

The Results section states that adjustment for BMI and anemia did not materially alter the association. However, the supplementary analyses indicate that referral status was also included in the adjusted models.

Please revise the Results sentence so that it consistently states adjustment for BMI, referral status, and anemia.

7. Improve the limitation regarding early-onset preeclampsia

The limitation currently states that the findings primarily apply to preeclampsia presenting at or beyond 28 weeks' gestation. However, based on the authors' own data, a notable proportion of participants were enrolled before 32 weeks and between 32–36 weeks.

Including this gestational-age distribution in the limitations section would improve transparency regarding the applicability of the findings across the preeclampsia spectrum.

8. Clarify the scope of the WHO recommendation

The cited WHO document appears to represent guidance from the Eastern Mediterranean Regional Office rather than a global WHO recommendation.

Please revise the wording to accurately reflect the scope of the cited guidance or replace it with the corresponding global WHO antenatal care guidance if that is the intended citation.

Finally, Before resubmission, I encourage the authors to perform one final cross-check of:

- sample sizes and denominators throughout the manuscript;

- frequencies and percentages across all tables;

- table and supplementary file numbering;

- figure captions and legends;

- in-text citations following reference renumbering; and

- consistency between the manuscript, supplementary materials, and submission metadata.

**********

what does this mean?). If published, this will include your full peer review and any attached files.

If you choose “no”, your identity will remain anonymous but your review may still be made public.

**Do you want your identity to be public for this peer review?**  For information about this choice, including consent withdrawal, please see our Privacy Policy

Reviewer #1: **Yes:** Georgios Iatrakis

Reviewer #2: **Yes:** IZERE Salomon

**********

[NOTE: If reviewer comments were submitted as an attachment file, they will be attached to this email and accessible via the submission site. Please log into your account, locate the manuscript record, and check for the action link "View Attachments". If this link does not appear, there are no attachment files.]

To ensure your figures meet our technical requirements, please review our figure guidelines: https://journals.plos.org/plosone/s/figures

You may also use PLOS’s free figure tool, NAAS, to help you prepare publication quality figures: https://journals.plos.org/plosone/s/figures#loc-tools-for-figure-preparation.

NAAS will assess whether your figures meet our technical requirements by comparing each figure against our figure specifications.

# Author comment: Author response to Decision Letter 2

**DOI:** 10.1371/journal.pone.0358781.r004

1 Sep 2026

Response to Reviewers

Manuscript number: PONE-D-26-14449

Manuscript title: Maternal Serum Vitamin D Levels and Preeclampsia among Kenyan Pregnant Women: A matched case-control study

Corresponding author: Kimbley Asaso Omwodo

________________________________________

We thank the Editor and both reviewers for their continued careful assessment of our manuscript. We are grateful to Reviewer #1 for confirming that the previous comments have been addressed. We have addressed each of Reviewer #2’s remaining points below and have made the corresponding changes in the revised manuscript, supporting information files, and submission metadata.

Reviewer #1

Comment: All comments have been addressed; the paper can now be published.

Response: We thank Reviewer #1 for their time and constructive feedback throughout the review process. We are pleased that the revisions are satisfactory.

Reviewer #2

We thank Reviewer #2 for acknowledging the improvements, and for identifying the remaining issues. We have addressed each point as follows.

Major Comment 1

Comment: Correct the remaining arithmetic inconsistency in Table 2 (Mandatory). The manuscript still contains one unresolved numerical inconsistency in Table 2. The control group vitamin D categories currently report: Deficient = 1, Insufficient = 3, Sufficient = 54. These values sum to 58, whereas the control sample size is reported as 59. Consequently, the overall total also sums to 117 instead of 118. Based on the reported suboptimal vitamin D counts elsewhere in the manuscript, the internally consistent values should be: - Controls: 55 participants (93.2%) with sufficient vitamin D. - Overall: 95 participants (80.5%) with sufficient vitamin D. Please correct Table 2 and the corresponding sentence in the Results section and verify that all percentages are recalculated using the correct denominators.

Response: We agree and apologise for the residual inconsistency. Table 2 has been corrected so that the control-group categories now read: deficient = 1 (1.7%), insufficient = 3 (5.1%), and sufficient = 55 (93.2%), summing to 59 controls. The overall row now reads: deficient = 6 (5.1%), insufficient = 17 (14.4%), and sufficient = 95 (80.5%), summing to 118 participants. The corresponding Results sentence has been updated to state that sufficient vitamin D levels (>=50 nmol/L) were present in 40 (67.8%) preeclampsia cases versus 55 (93.2%) controls. All percentages in Table 2 were recalculated using the corrected denominators.

Major Comment 2

Comment: Verify the originality argument after reference renumbering.

Response: We re-verified all in-text citations against the final, renumbered reference list. The novelty statement (final paragraph of the Introduction; first paragraph of the Discussion) is appropriately cautious, describing the study as 'context-specific' and an 'incremental contribution', and is supported by the cited African systematic review (Mogire et al., reference 13). This check identified one mis-citation introduced by the renumbering: the Endocrine Society guideline is now cited as reference 4 (previously reference 5).

Minor Comment 3

Comment: Ensure the Ethics Statement in the submission system matches the manuscript text by using MTRH and NACOSTI consistently.

Response: The Ethics Statement in the submission metadata has been updated to use 'MTRH' and 'NACOSTI' consistently, matching the manuscript text (IREC approval 0005019; NACOSTI licence NACOSTI/P/25/417821).

Minor Comment 4

Comment: Reconcile the Supporting Information file format.

Response: We have harmonised the Supporting Information: S1 File (completed STROBE checklist, PDF), S2 File (supplementary analyses, with tables relabelled S1–S5 Table), and S3 File (de-identified dataset, XLSX). The Supporting Information legend and all in-text citations have been updated accordingly.

Minor Comment 5

Comment: The manuscript states that vitamin D supplementation or multivitamin use exhibited "high collinearity with matching factors." This assertion is not supported by any statistical evidence presented in the manuscript. Unless supporting diagnostics are provided, I recommend replacing this statement with a simpler explanation indicating that supplementation was too infrequent to be modeled reliably. Also clarify that the supplementation denominator refers to the 124 enrolled participants, whereas the analytical sample consisted of 118 matched participants.

Response: We agree and have removed the unsupported collinearity statement. The revised text now states that vitamin D supplementation was too infrequent to model reliably: ‘Vitamin D supplementation was infrequent with 4 of 124 enrolled participants reporting any use. All were at less than 400 IU per day. Supplementation and multivitamin use could therefore not be modelled reliably as covariates. Any residual confounding from supplementation was likely minimal, as use was rare, low-dose, and similarly distributed between cases and controls.’

Minor Comment 6

Comment: Ensure consistency in the multivariable adjustment description. The Results section states that adjustment for BMI and anemia did not materially alter the association. However, the supplementary analyses indicate that referral status was also included in the adjusted models. Please revise the Results sentence so that it consistently states adjustment for BMI, referral status, and anemia.

Response: The Results sentence now consistently states that multivariable adjustment for BMI, referral status, and anemia did not materially alter the association between suboptimal vitamin D and preeclampsia. This wording matches the adjusted conditional logistic regression models presented in S2 File. We also clarified that complete-case adjusted models used smaller denominators because of missing BMI and haemoglobin data.

Minor Comment 7

Comment: The limitation currently states that the findings primarily apply to preeclampsia presenting at or beyond 28 weeks' gestation. However, based on the authors' own data, a notable proportion of participants were enrolled before 32 weeks and between 32–36 weeks.

Including this gestational-age distribution in the limitations section would improve transparency regarding the applicability of the findings across the preeclampsia spectrum.

Response: We have expanded the limitation to report the gestational-age distribution of the analytic cohort. ‘’Among the 118 matched participants, 16.9% were enrolled at <32 weeks and 38.1% at 32–36 weeks' gestation; moreover, all six participants excluded for lack of a match were <37 weeks. Our findings therefore apply mainly to PE presenting from 28 weeks onwards, are least informative for early-onset PE <32 weeks, and should be extrapolated to that subgroup with caution.’’

Minor Comment 8

Comment: Clarify whether the cited WHO guidance is regional or global.

Response: We agree the citation was regional guidance. We have replaced it with the global WHO antenatal care recommendations (WHO, 2016), which do not recommend universal vitamin D supplementation in pregnancy to prevent preeclampsia; the text now accurately reflects the scope of the cited guidance.

Final cross-check requested by Reviewer #2

Response: Before resubmission, we performed the requested final cross-check of: sample sizes and denominators throughout the manuscript; frequencies and percentages across all tables; table, figure, and supporting-information numbering; figure captions and legends; in-text citations after reference renumbering; and consistency among the manuscript, supplementary materials, and submission metadata.

Corrections arising from this cross-check are reflected in the revised manuscript and supporting files.

We hope that these revisions adequately address the remaining concerns and that the manuscript is now suitable for publication.

Sincerely,

The Authors

Attachment Submitted filename: Response_to_reviewers_auresp_2.docx

# Editor report: Decision Letter 2

**DOI:** 10.1371/journal.pone.0358781.r005

**Authors:** Maria Magnus

7 Sep 2026

Maternal Serum Vitamin D Levels and Preeclampsia among Kenyan Pregnant Women: A matched case-control study.

PONE-D-26-14449R2

Dear Dr. Asaso Omwodo,

We’re pleased to inform you that your manuscript has been judged scientifically suitable for publication and will be formally accepted for publication once it meets all outstanding technical requirements.

Within one week, you’ll receive an e-mail detailing the required amendments. When these have been addressed, you’ll receive a formal acceptance letter and your manuscript will be scheduled for publication.

An invoice will be generated when your article is formally accepted. Please note, if your institution has a publishing partnership with PLOS and your article meets the relevant criteria, all or part of your publication costs will be covered. Please make sure your user information is up-to-date by logging into Editorial Manager at Editorial Manager® and clicking the ‘Update My Information' link at the top of the page. For questions related to billing, please contact billing support.

If your institution or institutions have a press office, please notify them about your upcoming paper to help maximize its impact. If they’ll be preparing press materials, please inform our press team as soon as possible -- no later than 48 hours after receiving the formal acceptance. Your manuscript will remain under strict press embargo until 2 pm Eastern Time on the date of publication. For more information, please contact onepress@plos.org.

Kind regards,

Maria Christine Magnus, PhD

Academic Editor

PLOS One

Additional Editor Comments (optional):

Reviewers' comments:

# Editor report: Acceptance letter

**DOI:** 10.1371/journal.pone.0358781.r006

**Authors:** Maria Magnus

PONE-D-26-14449R2

PLOS One

Dear Dr. Asaso Omwodo,

I'm pleased to inform you that your manuscript has been deemed suitable for publication in PLOS One. Congratulations! Your manuscript is now being handed over to our production team.

At this stage, our production department will prepare your paper for publication. This includes ensuring the following:

* All references, tables, and figures are properly cited

* All relevant supporting information is included in the manuscript submission,

* There are no issues that prevent the paper from being properly typeset

You will receive further instructions from the production team, including instructions on how to review your proof when it is ready. Please keep in mind that we are working through a large volume of accepted articles, so please give us a few days to review your paper and let you know the next and final steps.

Lastly, if your institution or institutions have a press office, please let them know about your upcoming paper now to help maximize its impact. If they'll be preparing press materials, please inform our press team within the next 48 hours. Your manuscript will remain under strict press embargo until 2 pm Eastern Time on the date of publication. For more information, please contact onepress@plos.org.

You will receive an invoice from PLOS for your publication fee after your manuscript has reached the completed accept phase. If you receive an email requesting payment before acceptance or for any other service, this may be a phishing scheme. Learn how to identify phishing emails and protect your accounts at https://explore.plos.org/phishing.

If we can help with anything else, please email us at customercare@plos.org.

Thank you for submitting your work to PLOS One and supporting open access.

Kind regards,

PLOS One Editorial Office Staff

on behalf of

Dr. Maria Christine Magnus

Academic Editor

PLOS One
